# Supplementary material for: Systematic analyses of lipid mobilization by human lipid transfer proteins
Source: Nature. 2026 Jan 7;651(8105):511–20. doi: 10.1038/s41586-025-10040-y (PMC12979188; doi:10.1038/s41586-025-10040-y)
Supplement: Supplementary file 1 — This file contains Supplementary Figs. 1–4, Supplementary Tables 12–15, Methods and References [file 41586_2025_10040_MOESM1_ESM.pdf]

nature portfolio

https://doi.org/10.1038/s41586-025-10040-y

Supplementary information

Systematic analyses of lipid mobilization by human lipid transfer proteins

In the format provided by the authors and unedited

Supplementary information

Systematic analyses of lipid mobilization by human lipid transfer proteins

Kevin Titeca[1,2,#], Antonella Chiapparino[2,^#], Marco L. Hennrich[2,^^,#,*], Dénes Türei[3], Mahmoud Moqadam[4,5], Reza Talandashti[4,5,^^^], Camille Cuveillier[1], Larissa van Ek[1], Joanna Zukowska[2], Sergio Triana[2^^^^], Florian Echelard[4,5], Inger Ødum Nielsen[6], Mads Møller Foged[6], Charlotte Gehin[2,^^^^^], Kliment Olechnovic[7,8], Sergei Grudinin[8], Julio Saez-Rodriguez[3,9], Theodore Alexandrov[2, ^^^^^^], Kenji Maeda[6], Nathalie Reuter[4,5], Anne-Claude Gavin[1,*]

[1]*Department of Cell Physiology and Metabolism, University of Geneva, Geneva, Switzerland.*

[2]*European Molecular Biology Laboratory, EMBL, Heidelberg, Germany.*

[3]*Institute for Computational Biomedicine, Faculty of Medicine, Heidelberg University, and Heidelberg University Hospital, Heidelberg, Germany*.

[4]*Department of Chemistry, University of Bergen, Bergen, Norway.*

[5]*Computational Biology Unit, Department of Informatics, University of Bergen, Bergen, Norway.*

[6]*Cell Death and Metabolism group, Center for Autophagy, Recycling and Disease, Danish Cancer Institute, Copenhagen, Denmark.*

[7]*Institute of Biotechnology, Life Sciences Center, Vilnius University, Vilnius, Lithuania.*

[8]*CNRS, Grenoble INP, LJK, Université Grenoble Alpes, 38000 Grenoble, France.*

[9]*EMBL European Bioinformatics Institute (EMBL-EBI), Wellcome Genome Campus, Hinxton, UK.*

[^]*Current affiliation, AB Sciex Germany GmbH, Germany.*

[^^]*Current affiliation, Absea Biotechnology GmbH Berlin, Germany.*

[^^^]*Current affiliation, Department of Biochemistry, University of Oxford, Oxford, UK.*

[^^^^]*Current affiliation, Institute for Medical Engineering and Science (IMES) and Department of Chemistry, Massachusetts Institute of Technology, Cambridge, MA, USA; Broad Institute of MIT and Harvard, Cambridge, MA, USA*

[^^^^^]*Current affiliation: École Polytechnique Fédérale de Lausanne (EPFL), Switzerland*.

[^^^^^^]*Current affiliation: Department of Pharmacology, University of California San Diego, La Jolla, CA 92093, USA; DeepCyte Inc., San Diego, CA 92122, USA.*

# These authors contributed equally:

**Correspondence and requests for materials E-mail: anne-claude.gavin@unige.ch and marco.hennrich@abseabio.com.*

Supplementary Information

Supplementary Figures 2

Supplementary Fig. 1 2

Supplementary Fig. 2 20

Supplementary Fig. 3 21

Supplementary Fig. 4 22

Supplementary Tables 23

Supplementary Table 12 23

Supplementary Table 13 24

Supplementary Table 14 25

Supplementary Table 15 26

Supplementary Methods 27

References 45

Supplementary Figures

Supplementary Figure 1a: Purification and characterization of LTPs-lipid complexes *in cellulo* (with SEC, SDS-PAGE and HPTLC data). LTP-lipid complexes were isolated from the respective cell lines and fractionated on Superdex 200 SEC column. Upper panels, chromatogram (A215). Middle panels: SDS-PAGE of purified LTPs. Lower Panel, HPTLC analysis of GPL, SL and sterols of LTPs isolated from the respective human cell lines. On top of each chromatogram, the resolution of the column is displayed. **a,** OSBP domain proteins**. b,** START domain proteins. **c,** ML domain protein. **d,** CRAL-TRIO domain proteins. **e**, SDS-PAGE and HTPLC are shown for the START domain protein, PCTP (synonym of STARD2) and for the GLTP domain protein, GLTP. These two proteins were fractionated on a different SEC column.
*The indicated region of the HPTLC is displayed with enhanced contrast below or next the full HPTLC image. S, standards: MW markers for SDS-PAGE and lipid standards for HPTLC; E, elution. PIPs, phosphatidylinositol phosphate; PC, phosphatidylcholine; PI, phosphatidylinositol; PS, phosphatidylserine; PA, phosphatidic acid; CA, cardiolipin; PE, phosphatidylethanolamine; Chol, cholesterol; CO, cholesteryl oleate; SphP, sphingosine-1-phosphate; SM, sphingomyelin; CerP, ceramide-1-phosphate; Cer, ceramide; S, standard containing GPL, cholesterol and CO; S2, standard containing SL (see CERT binding profile, panel **d**). S3 contains Erg (ergosterol) instead of cholesterol and does not contain CO (only used for experiments in panel **e**).

a

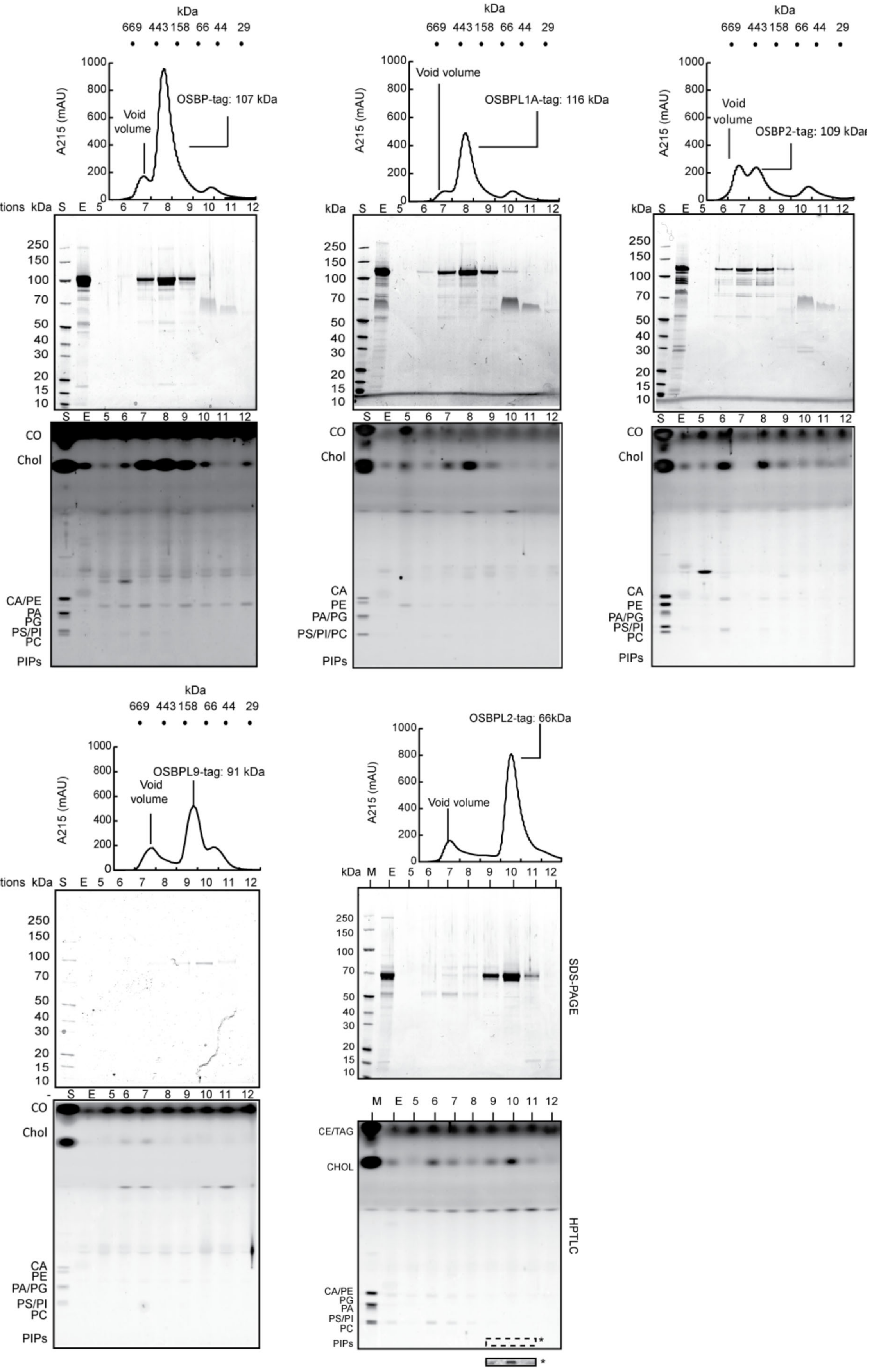

kDa
669 443 158 66 44 29
A215 (mAU)
Void volume
OSBP-tag: 107 kDa
OSBPL1A-tag: 116 kDa
OSBP2-tag: 109 kDa
OSBPL9-tag: 91 kDa
OSBPL2-tag: 66kDa
Fractions kDa S E 5 6 7 8 9 10 11 12
CO
Chol
CA/PE
PA
PG
PS/PI
PC
PIPs
CA
PE
PA/PG
PS/PI/PC
CE/TAG
CHOL
SDS-PAGE
HPTLC

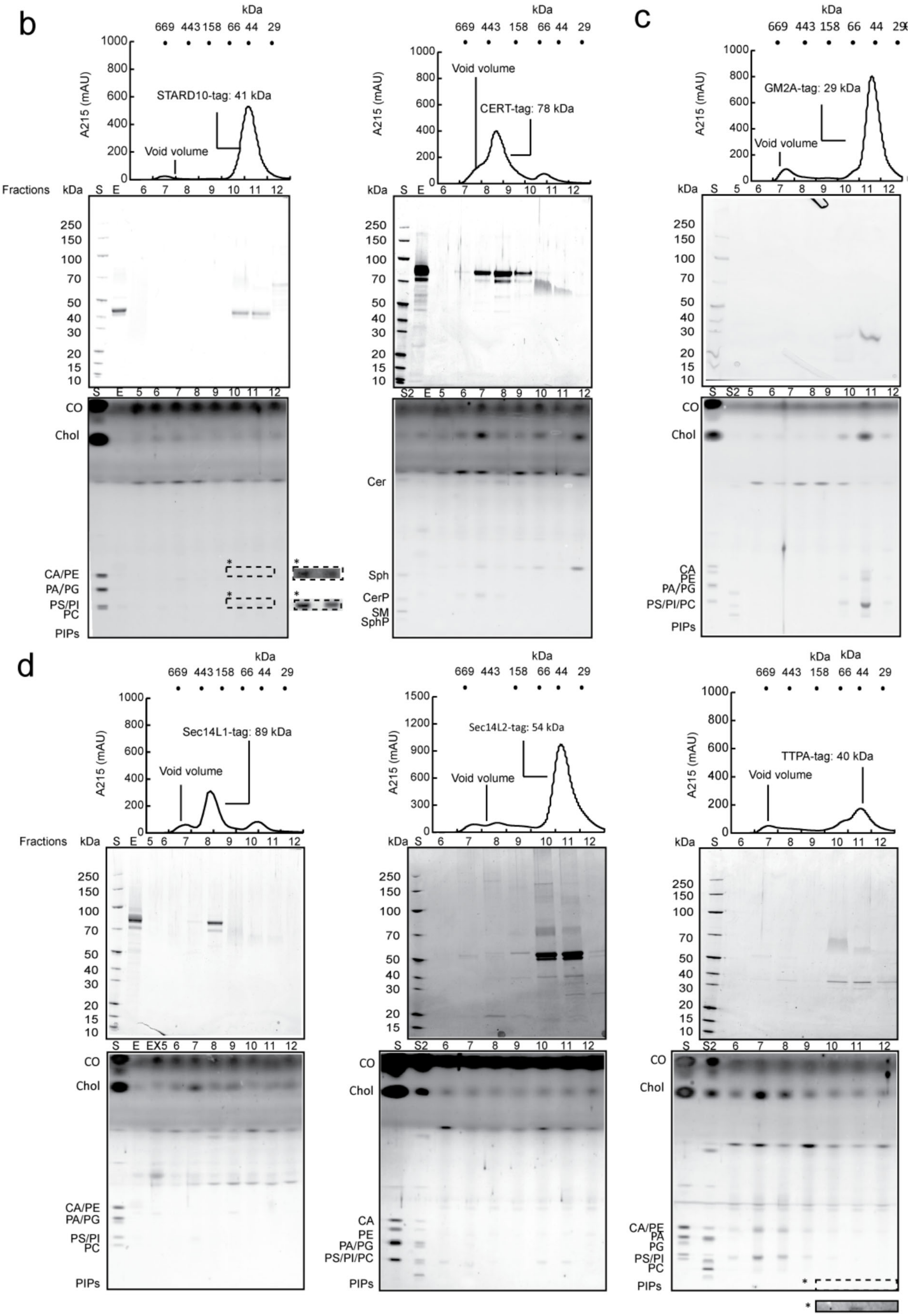
b
kDa
669 443 158 66 44 29
A215 (mAU)
STARD10-tag: 41 kDa
Void volume
Fractions kDa S E 6 7 8 9 10 11 12
250
150
100
70
50
40
30
20
15
10
S E 5 6 7 8 9 10 11 12
CO
Chol
CA/PE
PA/PG
PS/PI
PC
PIPs
CERT-tag: 78 kDa
S E 6 7 8 9 10 11 12
S2 E 5 6 7 8 9 10 11 12
Cer
Sph
CerP
SM
SphP
c
GM2A-tag: 29 kDa
S 5 6 7 8 9 10 11 12
S S2 5 6 7 8 9 10 11 12
CA
PE
PA/PG
PS/PI/PC
d
Sec14L1-tag: 89 kDa
S E 5 6 7 8 9 10 11 12
S E EX5 6 7 8 9 10 11 12
Sec14L2-tag: 54 kDa
S 6 7 8 9 10 11 12
S S2 6 7 8 9 10 11 12
PA/PG
PS/PI/PC
TTPA-tag: 40 kDa
S S2 6 7 8 9 10 11 12
PA
PG

e

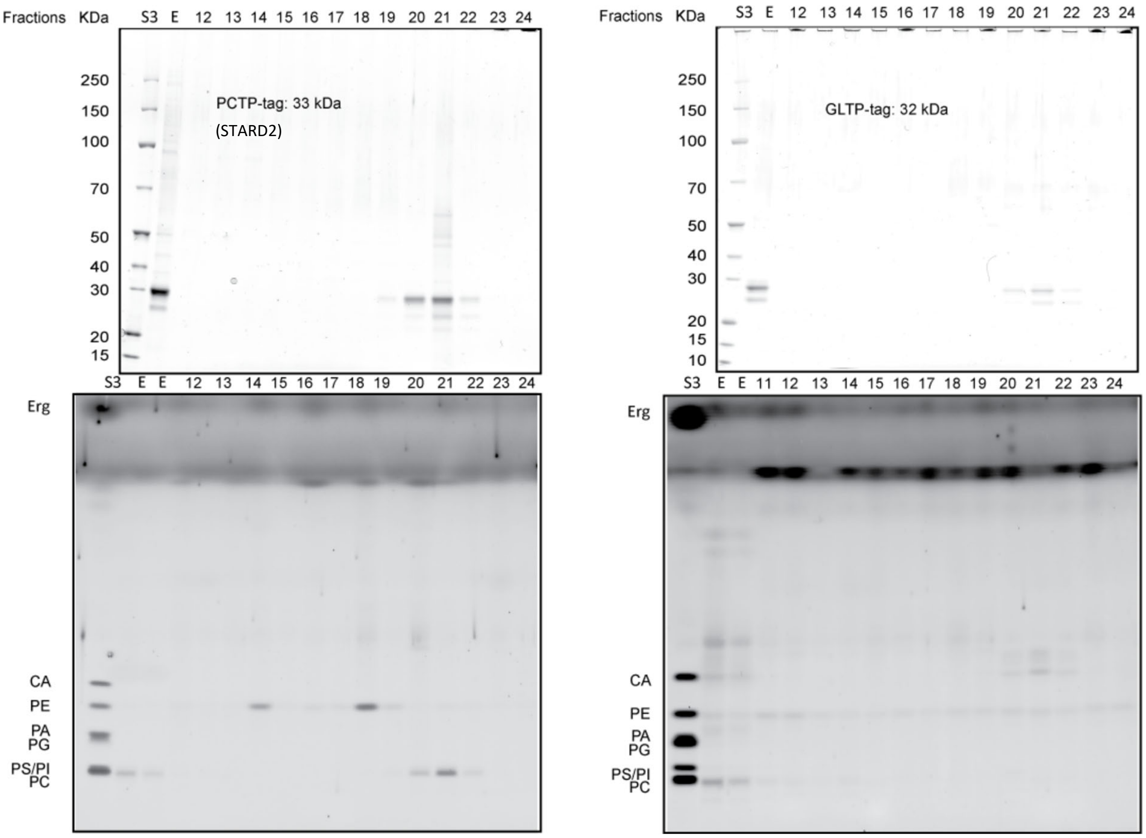
Fractions KDa S3 E 12 13 14 15 16 17 18 19 20 21 22 23 24
250
150
100
70
50
40
30
20
15
PCTP-tag: 33 kDa
(STARD2)
S3 E E 12 13 14 15 16 17 18 19 20 21 22 23 24
Erg
CA
PE
PA
PG
PS/PI
PC
Fractions KDa S3 E 12 13 14 15 16 17 18 19 20 21 22 23 24
250
150
100
70
50
40
30
20
15
10
GLTP-tag: 32 kDa
S3 E E 11 12 13 14 15 16 17 18 19 20 21 22 23 24
Erg
CA
PE
PA
PG
PS/PI
PC

Purification and characterization of LTPs-lipid complexes *in cellulo* (with SEC and SDS-PAGE data)

Upper panels: Chromatogram (A215). Lower panels: SDS-PAGE of LTPs isolated from the respective human cell lines. On top of each chromatogram, the resolution of the column is displayed. **a,** OSBP domain proteins. **b,** lipocalin domain proteins. **c,** CRAL-TRIO domain proteins. **d,** SCP2 domain protein; **e,** PITP domain proteins. **f,** GLTP domain protein. **g,** NPC1 NTD domain protein. **h,** START domain proteins. **i,** LBP_BPI_CETP domain proteins. S, standards, MW markers; E, elution.

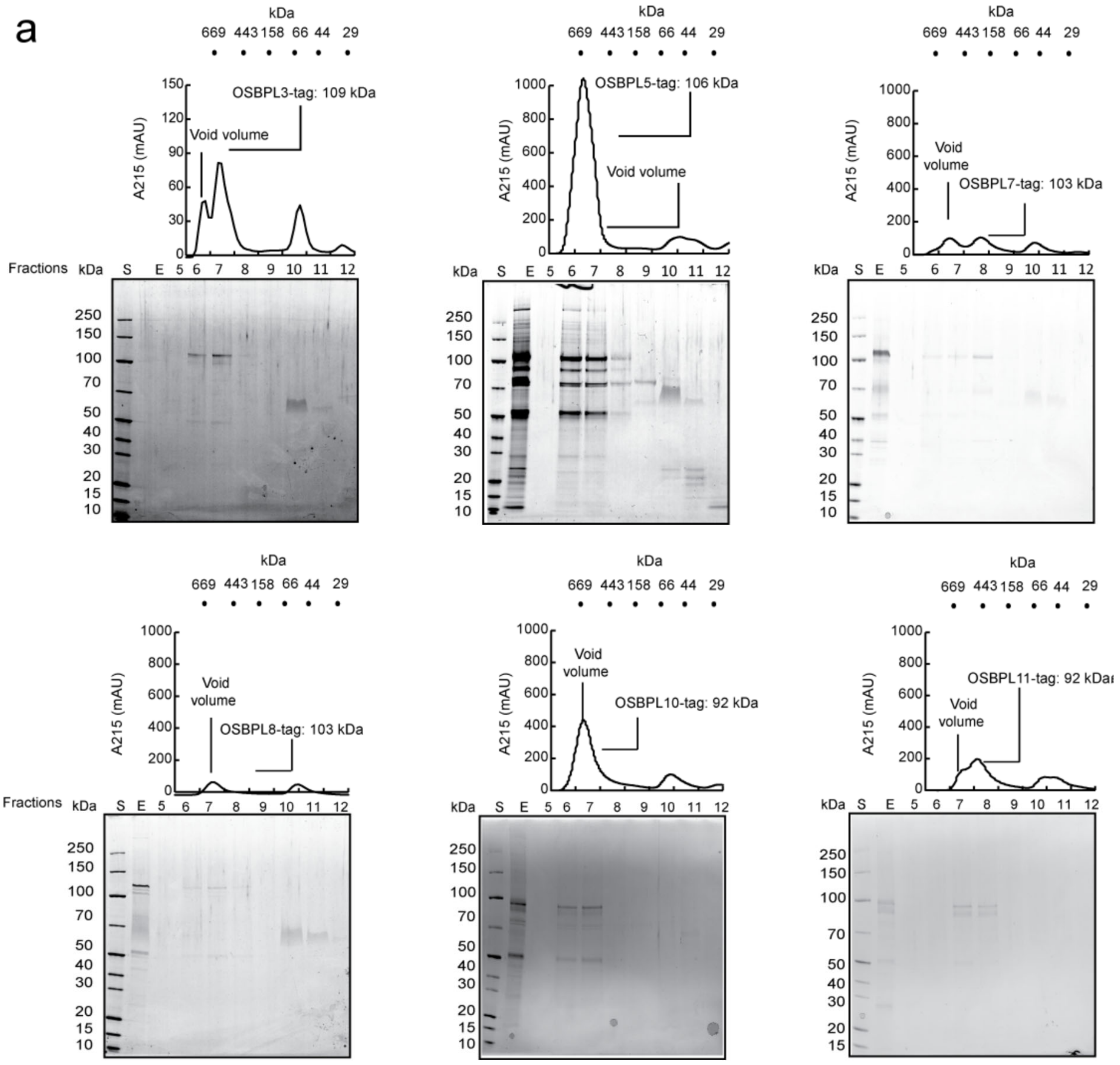

b

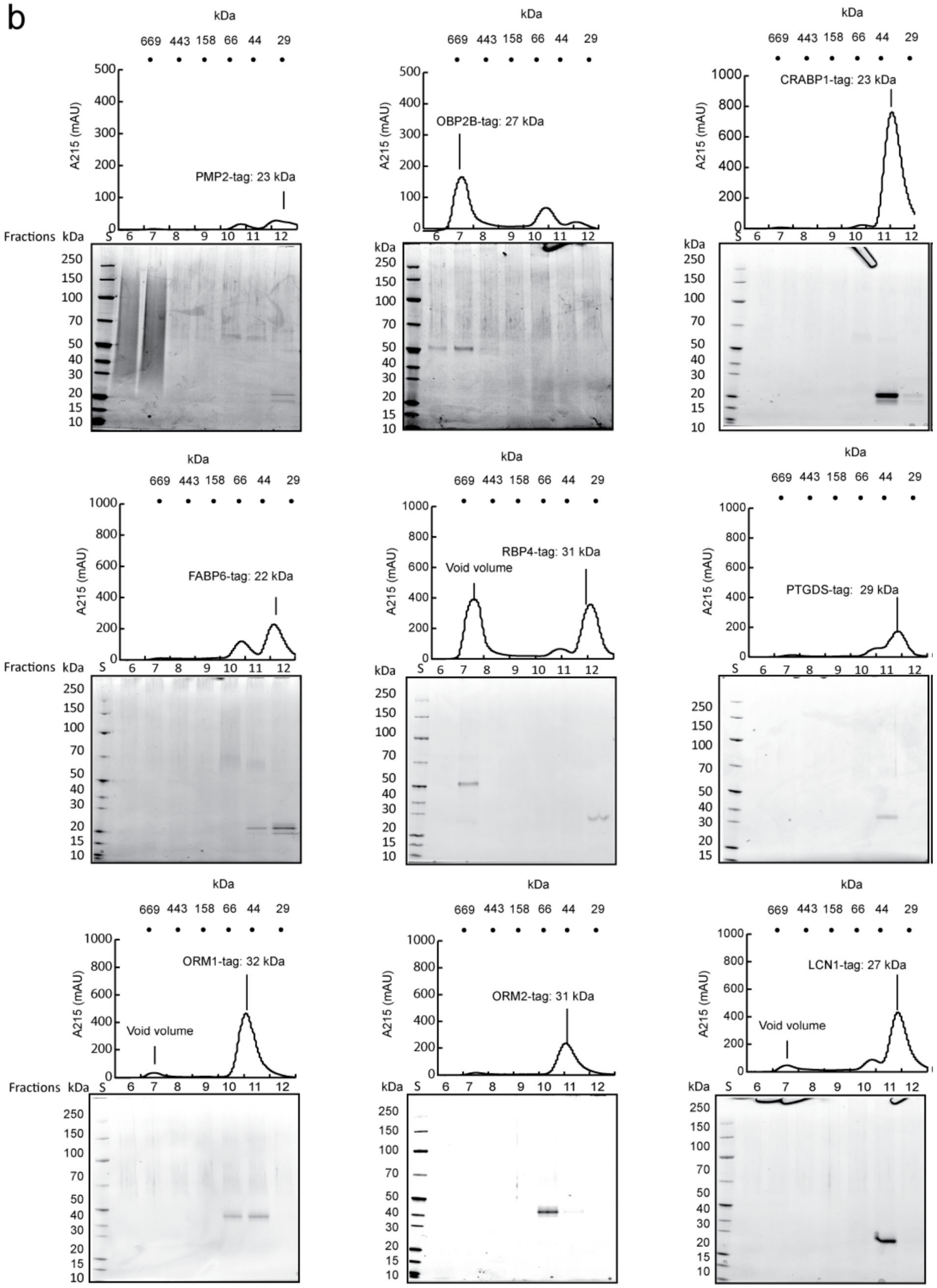
kDa
669 443 158 66 44 29
A215 (mAU)
PMP2-tag: 23 kDa
OBP2B-tag: 27 kDa
CRABP1-tag: 23 kDa
FABP6-tag: 22 kDa
RBP4-tag: 31 kDa
Void volume
PTGDS-tag: 29 kDa
ORM1-tag: 32 kDa
ORM2-tag: 31 kDa
LCN1-tag: 27 kDa
Fractions kDa S 6 7 8 9 10 11 12
250 150 100 70 50 40 30 20 15 10

C

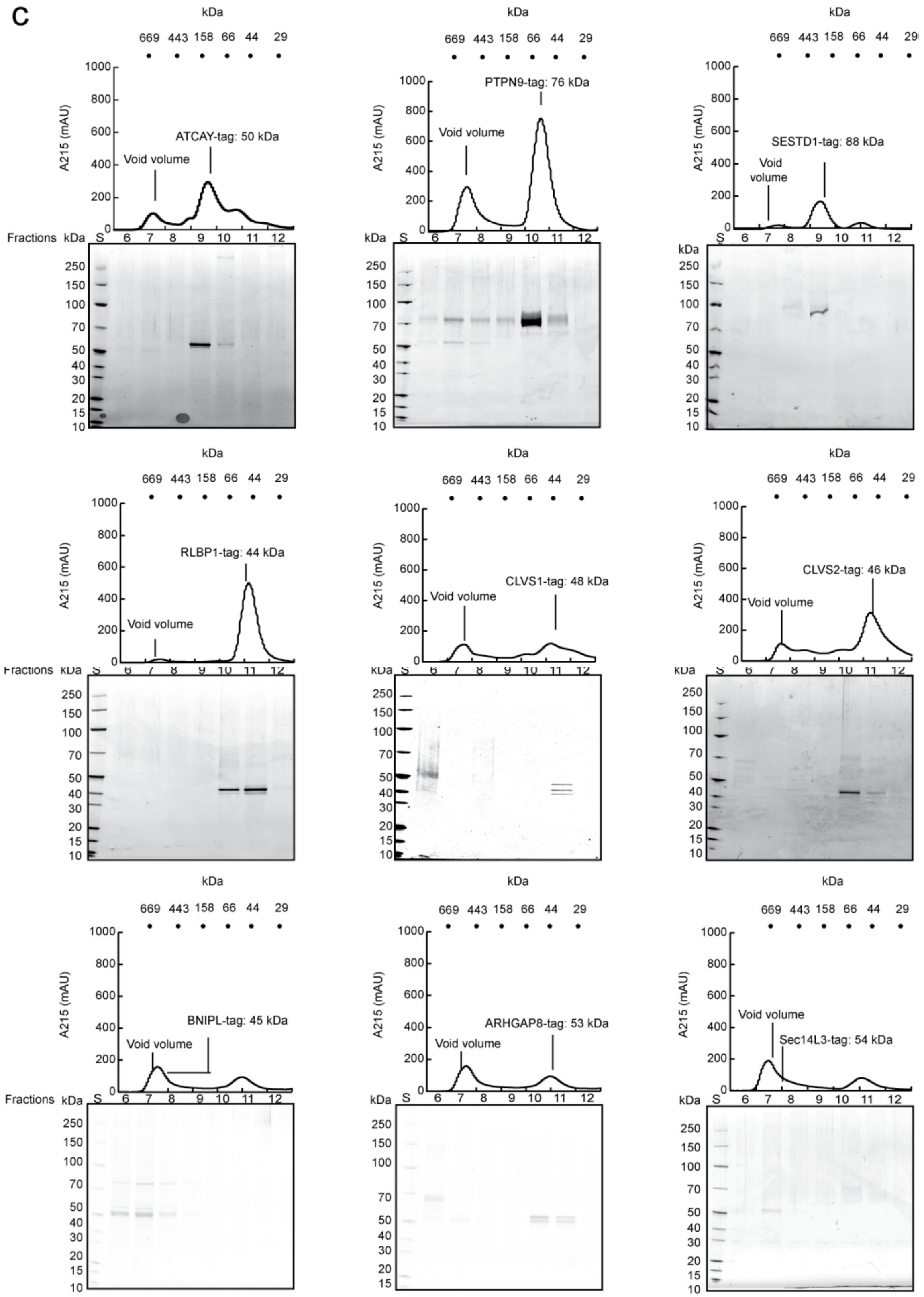
kDa
669 443 158 66 44 29
A215 (mAU)
Void volume
ATCAY-tag: 50 kDa
PTPN9-tag: 76 kDa
SESTD1-tag: 88 kDa
RLBP1-tag: 44 kDa
CLVS1-tag: 48 kDa
CLVS2-tag: 46 kDa
BNIPL-tag: 45 kDa
ARHGAP8-tag: 53 kDa
Sec14L3-tag: 54 kDa
Fractions
S 6 7 8 9 10 11 12
250 150 100 70 50 40 30 20 15 10

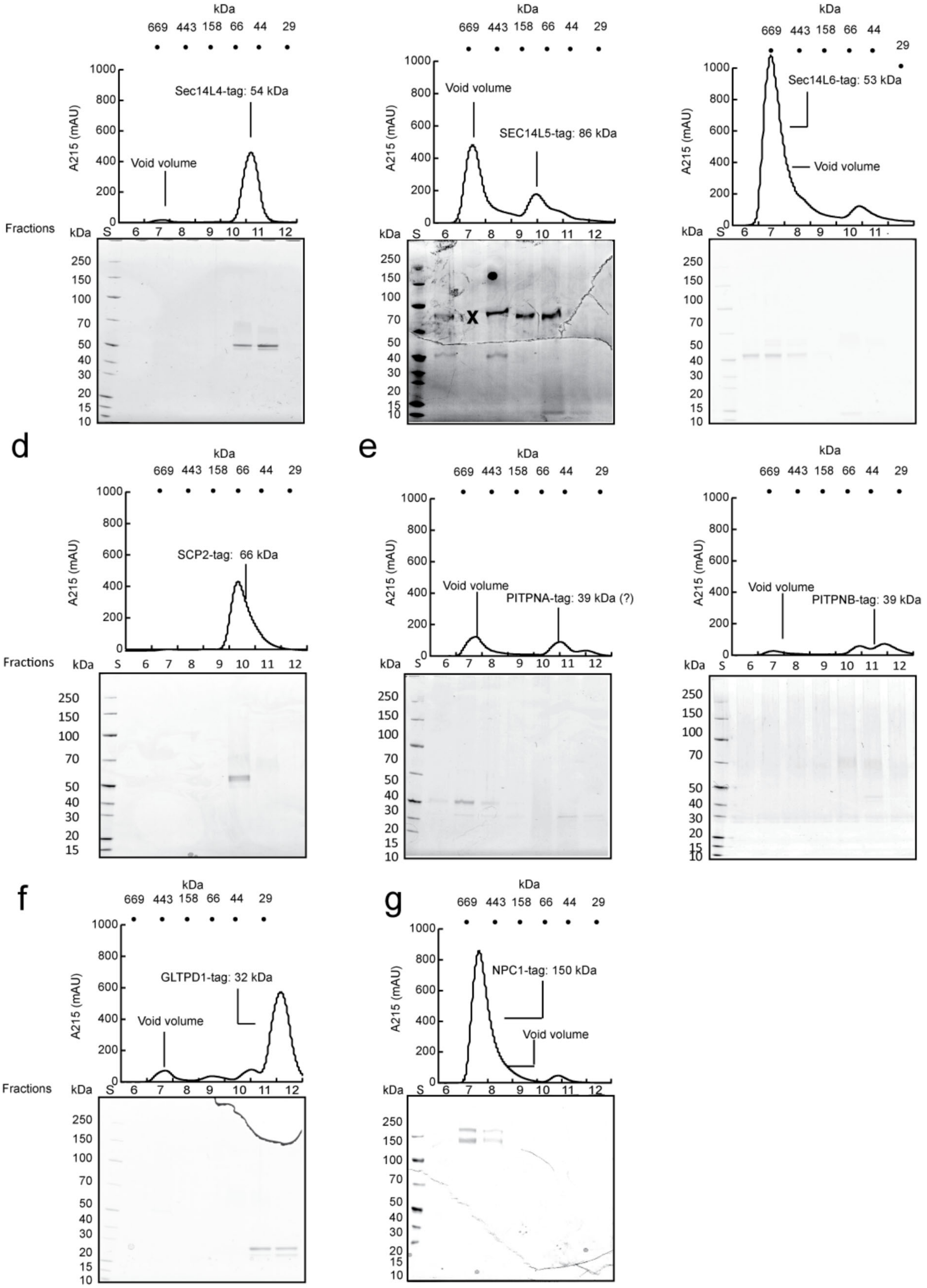
kDa
669 443 158 66 44 29
A215 (mAU)
Sec14L4-tag: 54 kDa
Void volume
Fractions
SEC14L5-tag: 86 kDa
Sec14L6-tag: 53 kDa
d
SCP2-tag: 66 kDa
e
PITPNA-tag: 39 kDa (?)
PITPNB-tag: 39 kDa
f
GLTPD1-tag: 32 kDa
g
NPC1-tag: 150 kDa

h

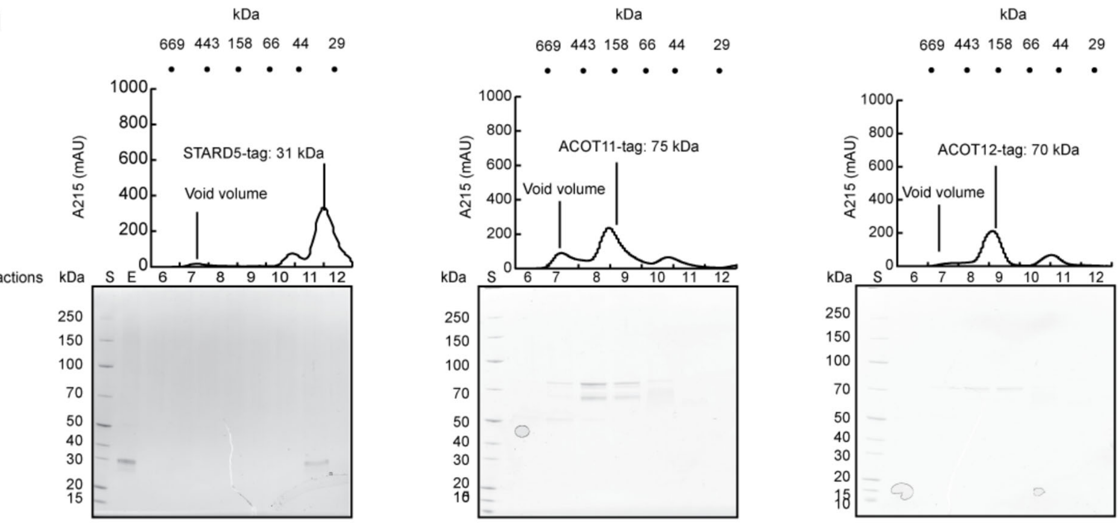

kDa
669 443 158 66 44 29
A215 (mAU)
STARD5-tag: 31 kDa
Void volume
Fractions kDa S E 6 7 8 9 10 11 12
250 150 100 70 50 40 30 20 15
ACOT11-tag: 75 kDa
Void volume
kDa S 6 7 8 9 10 11 12
250 150 100 70 50 40 30 20 15
ACOT12-tag: 70 kDa
Void volume
kDa S 6 7 8 9 10 11 12
250 150 100 70 50 40 30 20 15 10

i

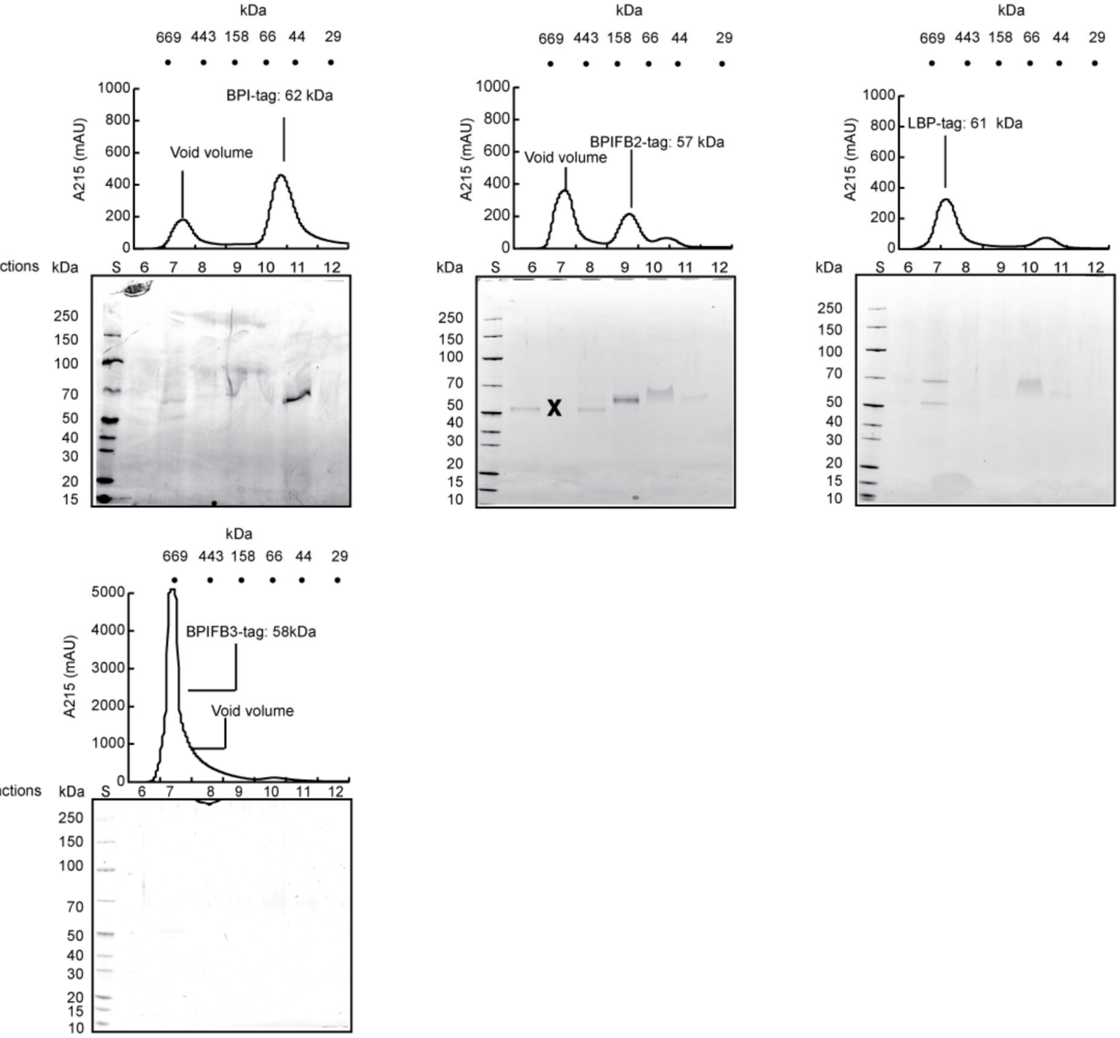

kDa
669 443 158 66 44 29
A215 (mAU)
BPI-tag: 62 kDa
Void volume
Fractions kDa S 6 7 8 9 10 11 12
250 150 100 70 50 40 30 20 15
BPIFB2-tag: 57 kDa
Void volume
kDa S 6 7 8 9 10 11 12
250 150 100 70 50 40 30 20 15 10
X
LBP-tag: 61 kDa
kDa S 6 7 8 9 10 11 12
250 150 100 70 50 40 30 20 15 10
BPIFB3-tag: 58kDa
Void volume
Fractions kDa S 6 7 8 9 10 11 12
250 150 100 70 50 40 30 20 15 10

Supplementary Figure 1b:

Purification and characterization of LTPs-lipid complexes *in vitro* (SEC results): Size exclusion chromatography profiles of *in vitro* purified LTP-lipid complexes. After elution from the affinity column, the LTP-lipid complexes were isolated on a Superdex 200 size exclusion resin packed in a Tricorn 5/150 Column (GE Healthcare).

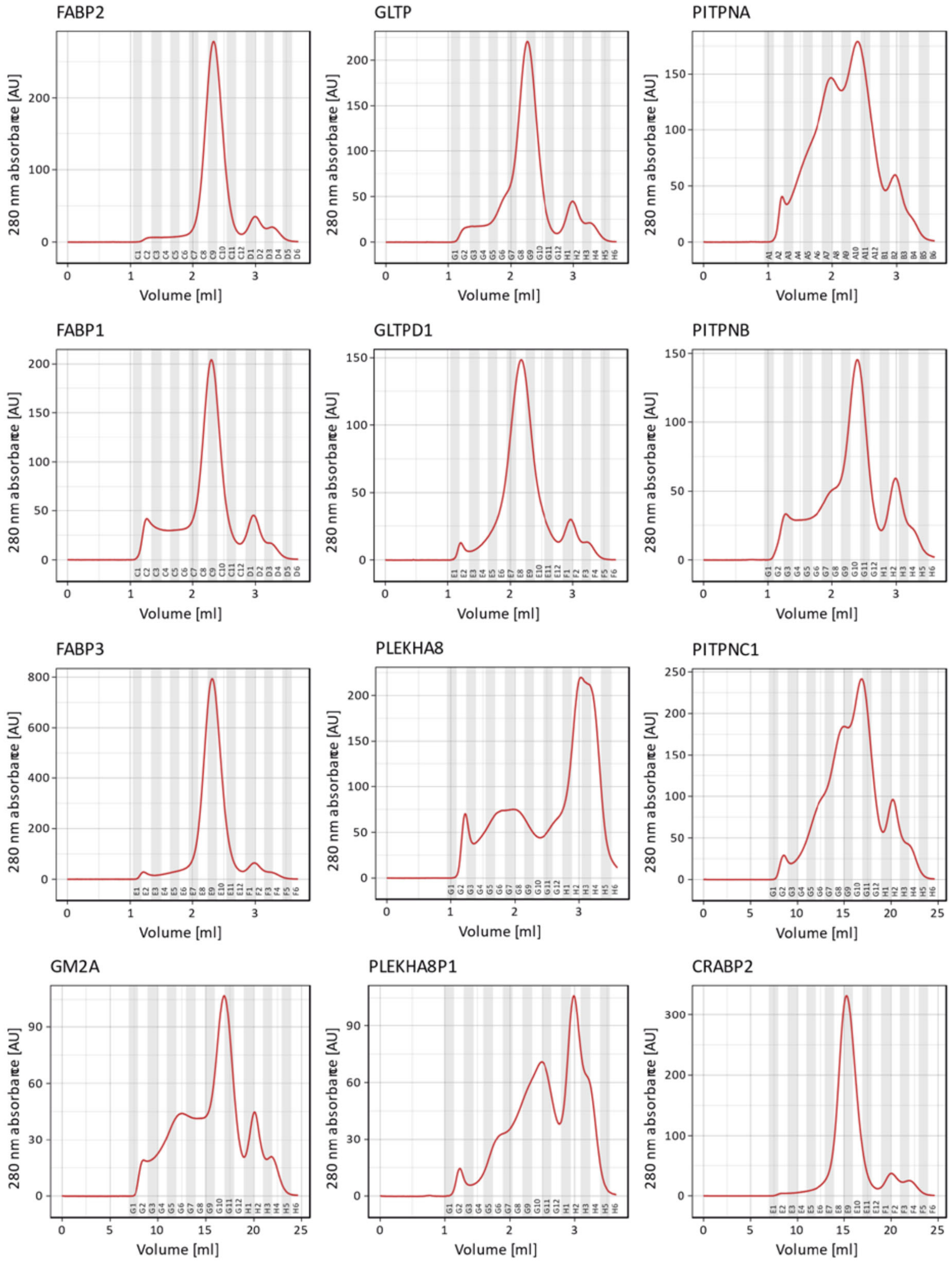

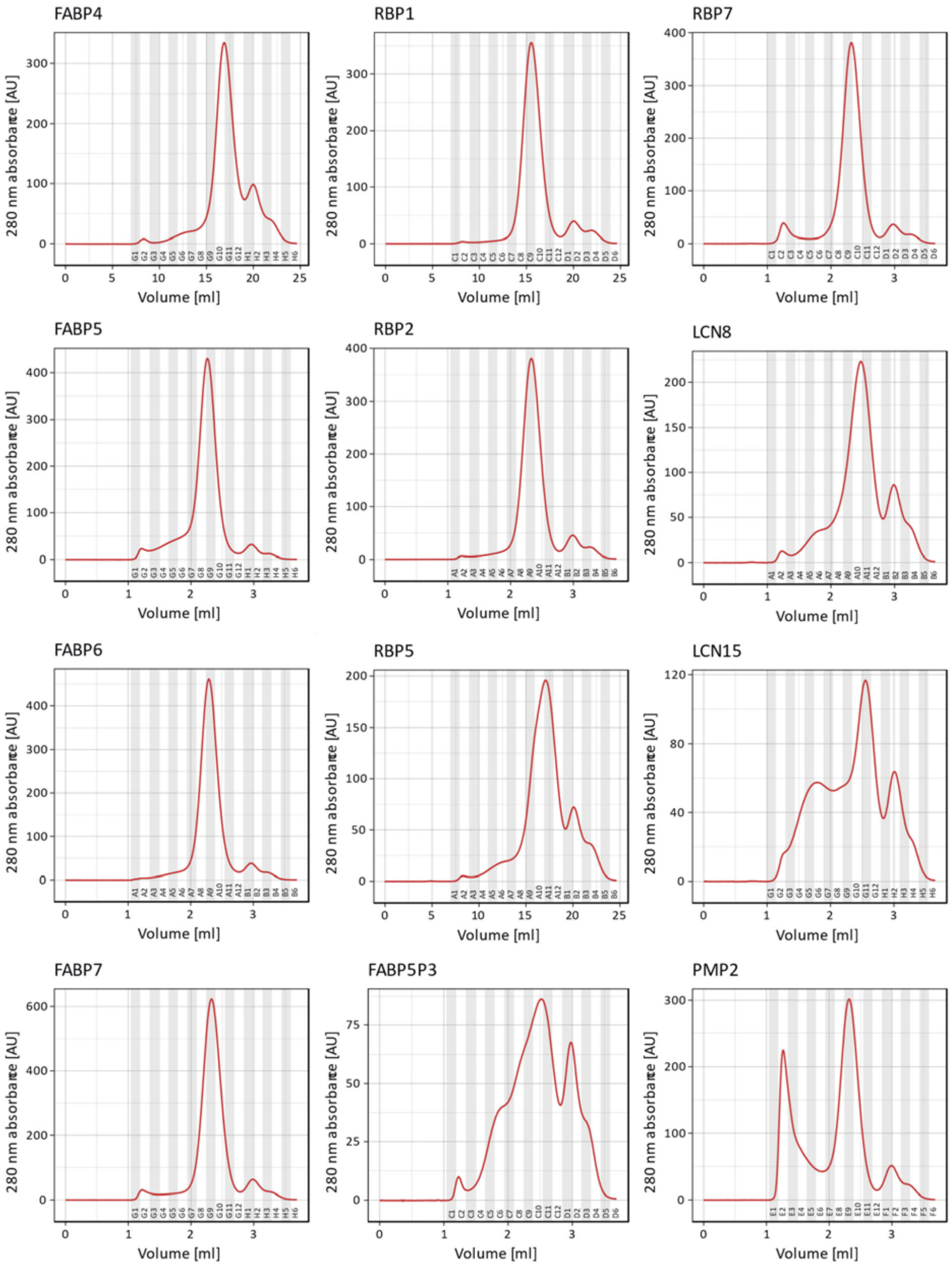
FABP4
RBP1
RBP7
FABP5
RBP2
LCN8
FABP6
RBP5
LCN15
FABP7
FABP5P3
PMP2
280 nm absorbance [AU]
Volume [ml]

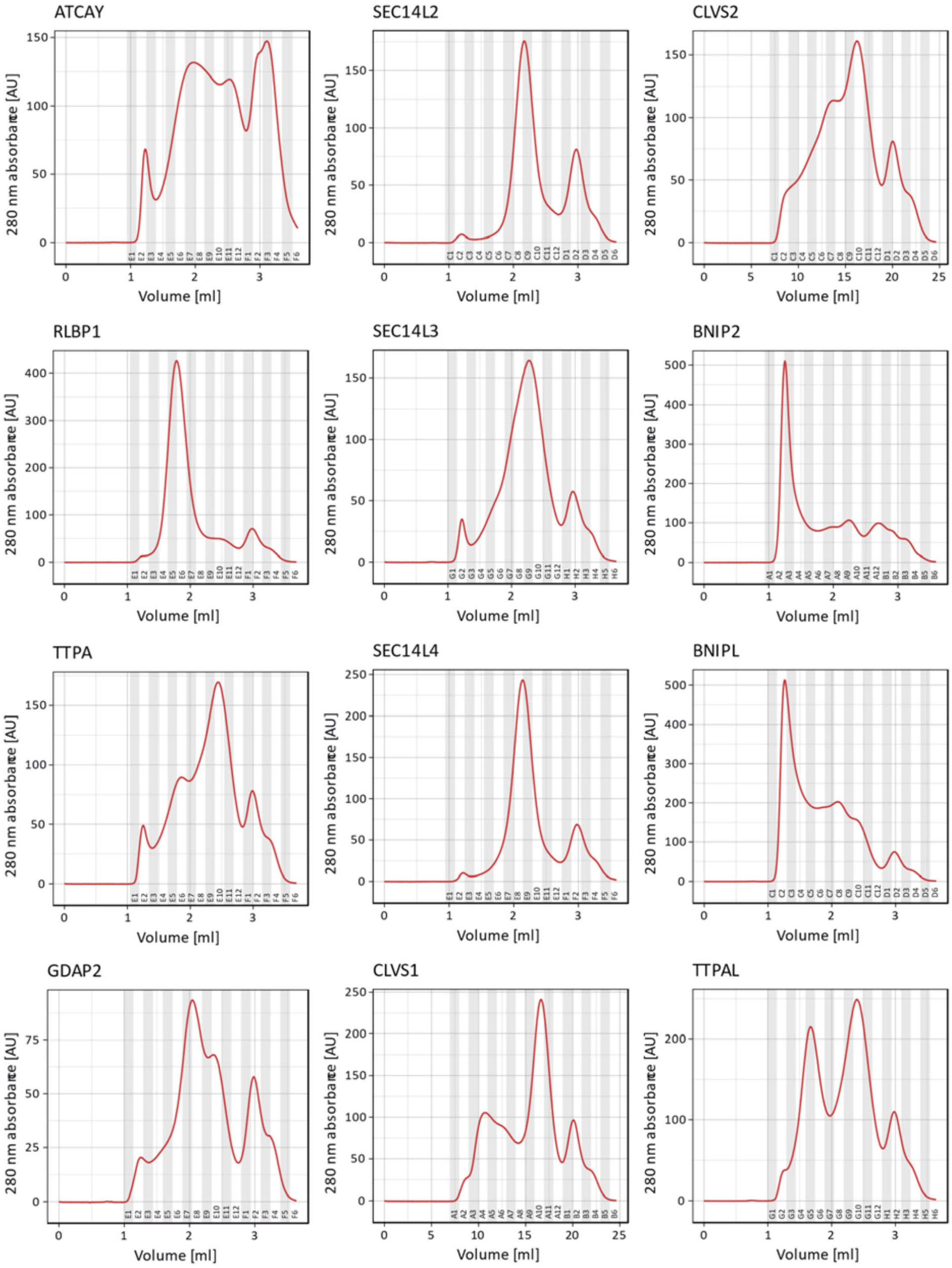

ATCAY
SEC14L2
CLVS2
RLBP1
SEC14L3
BNIP2
TTPA
SEC14L4
BNIPL
GDAP2
CLVS1
TTPAL
280 nm absorbance [AU]
Volume [ml]

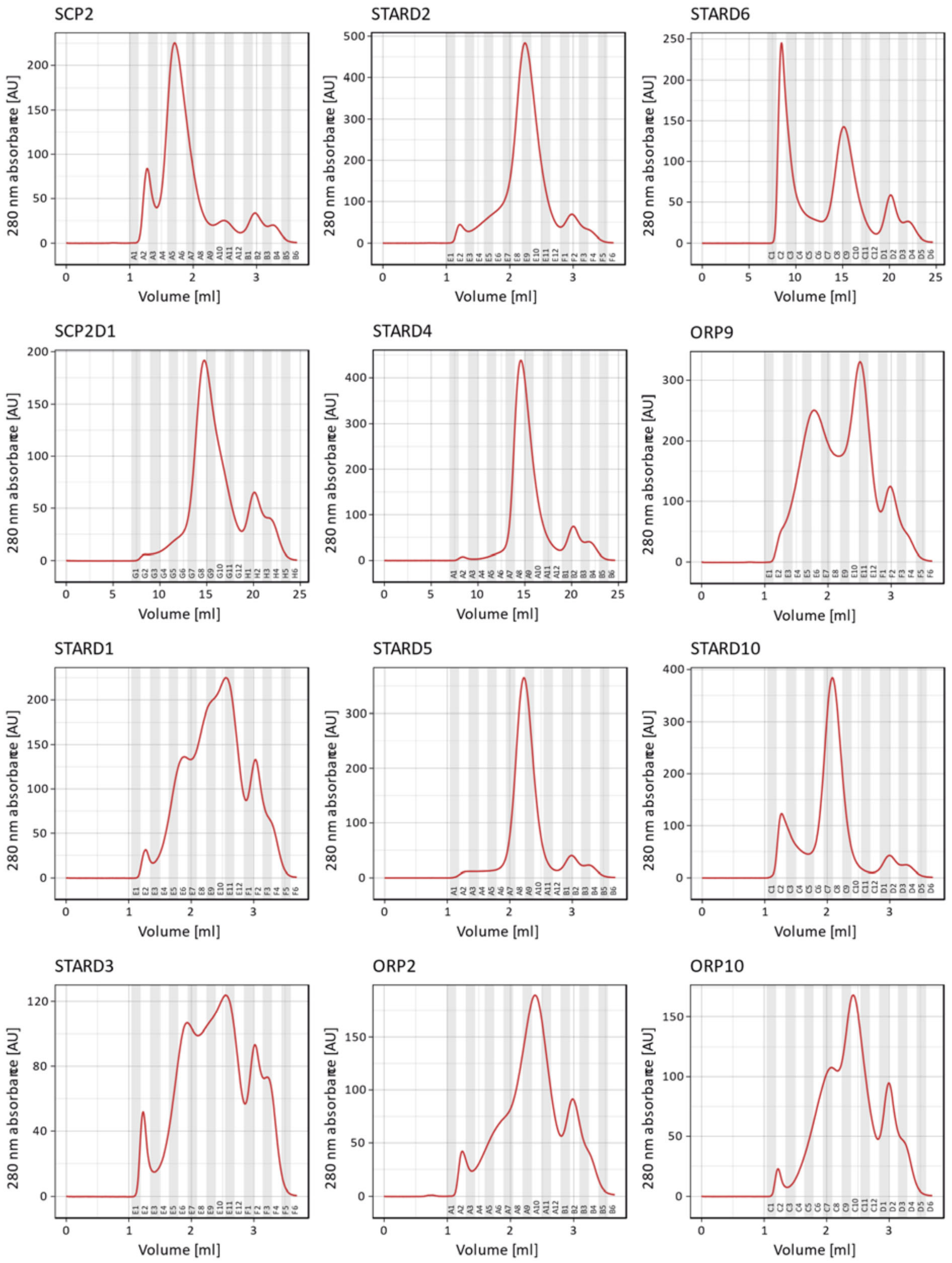
SCP2
STARD2
STARD6
SCP2D1
STARD4
ORP9
STARD1
STARD5
STARD10
STARD3
ORP2
ORP10
280 nm absorbance [AU]
Volume [ml]

Purification and characterization of LTPs-lipid complexes *in vitro* (SDS-PAGE results):
SDS-PAGE analysis of SEC fractions of *in vitro* purified LTP-lipid complexes. After elution from the affinity column, the LTP-lipid complexes were isolated on a Superdex 200 size exclusion resin packed in a Tricorn 5/150 Column (GE Healthcare). Proteins gels are stained in colloidal Coomassie Blue.

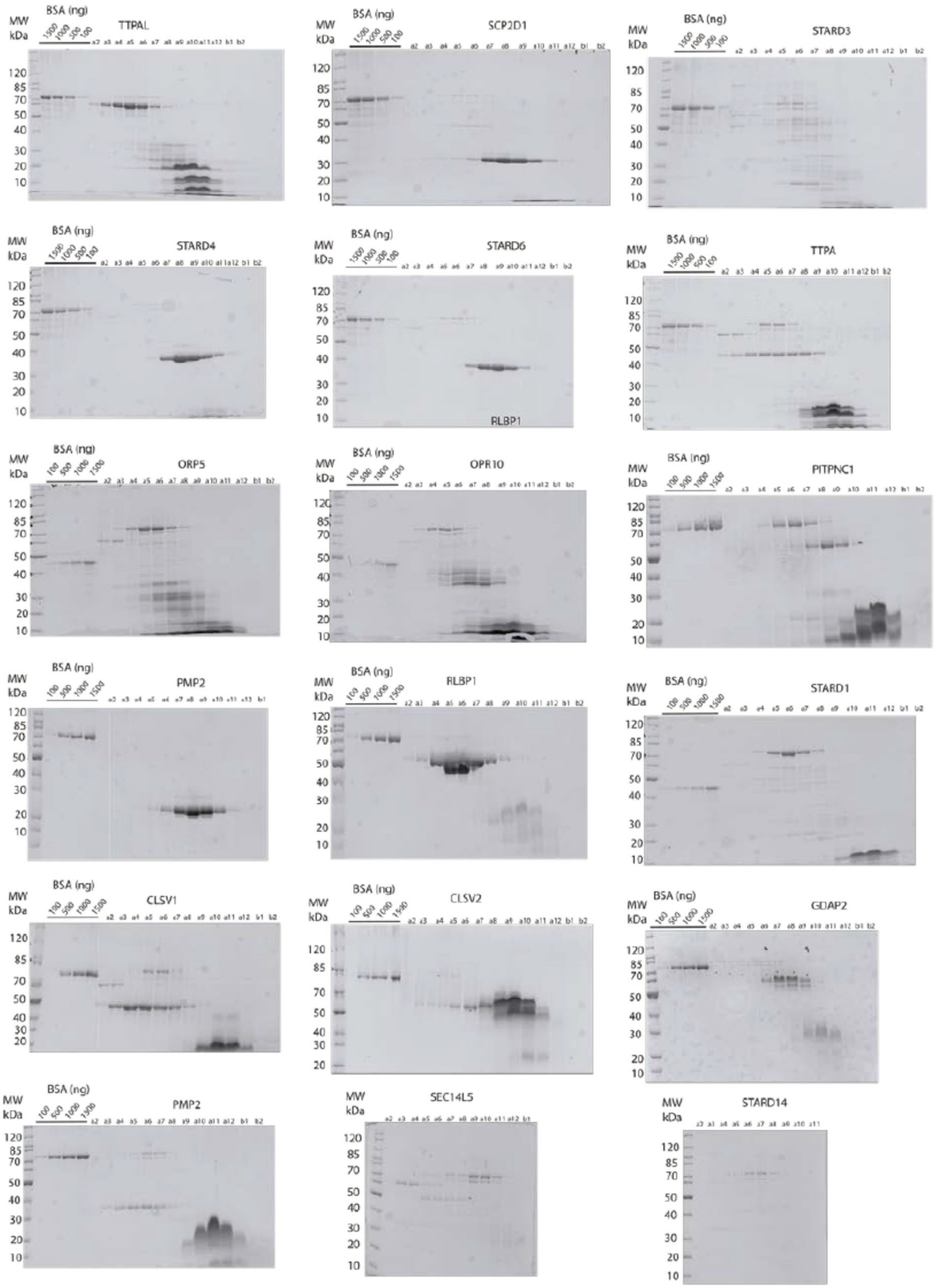

Supplementary Figure 1c: Overexpression of the N- and C-terminally tagged LTPs in the HEK-LTP cell lines.

Shown are the western blot images of the paired biological HEK-LTP cell line triplicates incubated with anti-HA antibody and visualised with ECL. The blots are alphabetically ordered based on the LTP name in the construct. Non-induced (-) and 72 hour-tetracycline-induced (+) biological triplicates (A, B, C) for each HEK-LTP cell line are compared side to side. At the bottom of the blot is the exposure time of the blot in the imager indicated. The arrows besides the blots indicates the correct height of the N- or C- terminally tagged HA-6xHis-2xStrepTagII-tagged LTP. After transfer, the molecular weight standards were visualized on the Western Blot membranes by Ponceau staining. Their positions are overlaid on the left side of the Western blot.

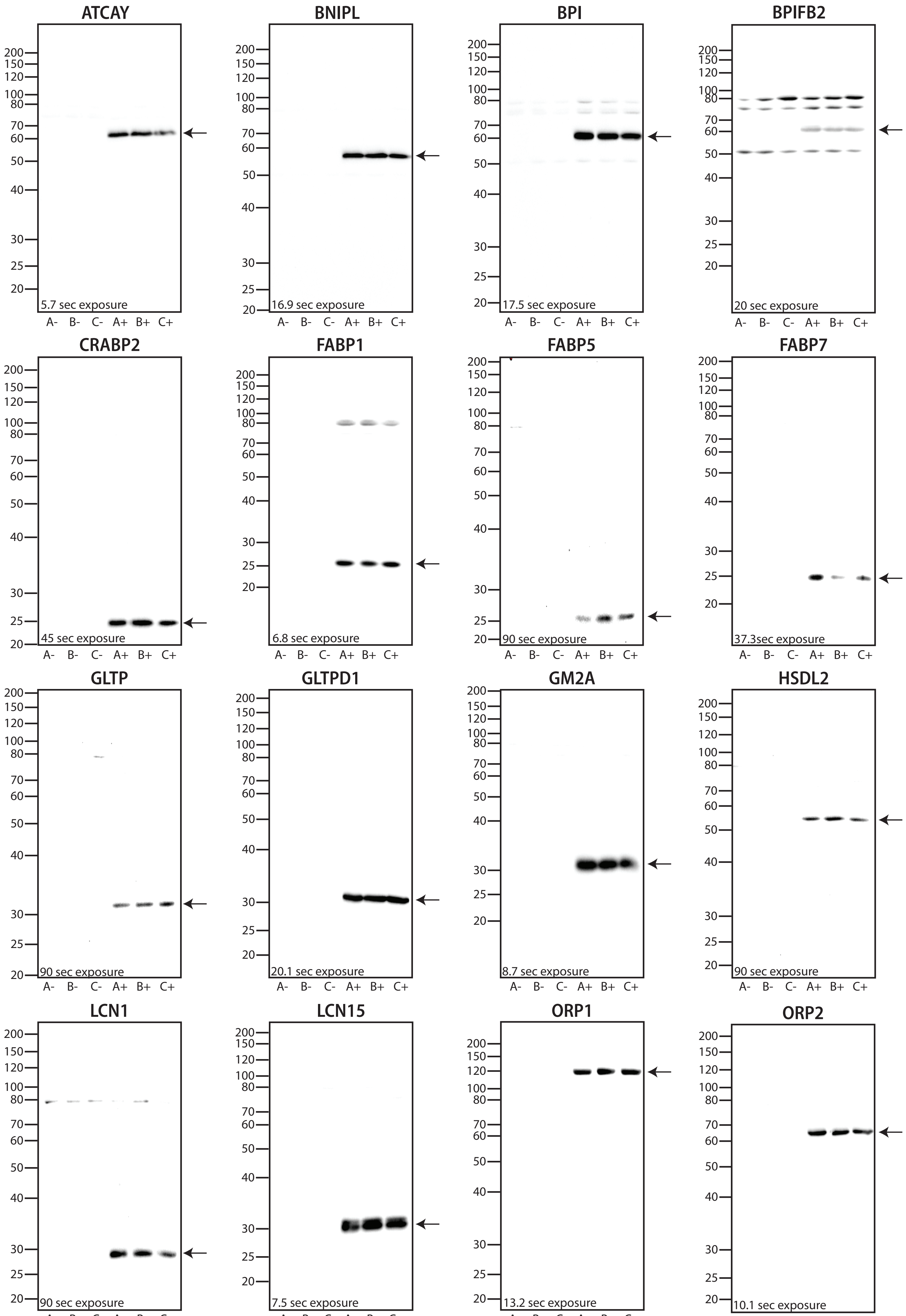
ATCAY
5.7 sec exposure
BNIPL
16.9 sec exposure
BPI
17.5 sec exposure
BPIFB2
20 sec exposure
CRABP2
45 sec exposure
FABP1
6.8 sec exposure
FABP5
90 sec exposure
FABP7
37.3sec exposure
GLTP
90 sec exposure
GLTPD1
20.1 sec exposure
GM2A
8.7 sec exposure
HSDL2
90 sec exposure
LCN1
90 sec exposure
LCN15
7.5 sec exposure
ORP1
13.2 sec exposure
ORP2
10.1 sec exposure
A- B- C- A+ B+ C+

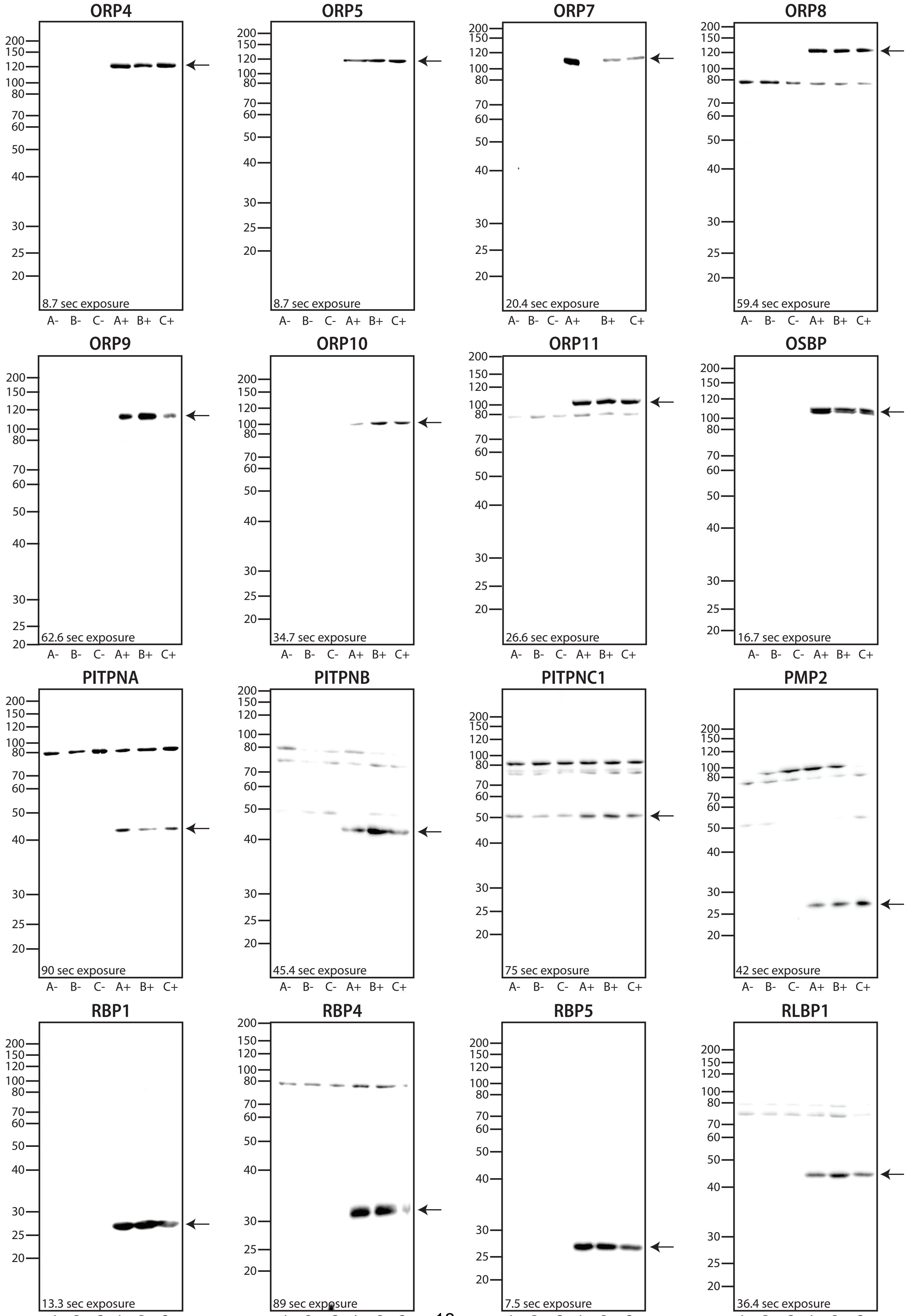
ORP4
8.7 sec exposure
ORP5
8.7 sec exposure
ORP7
20.4 sec exposure
ORP8
59.4 sec exposure
ORP9
62.6 sec exposure
ORP10
34.7 sec exposure
ORP11
26.6 sec exposure
OSBP
16.7 sec exposure
PITPNA
90 sec exposure
PITPNB
45.4 sec exposure
PITPNC1
75 sec exposure
PMP2
42 sec exposure
RBP1
13.3 sec exposure
RBP4
89 sec exposure
RBP5
7.5 sec exposure
RLBP1
36.4 sec exposure
A- B- C- A+ B+ C+

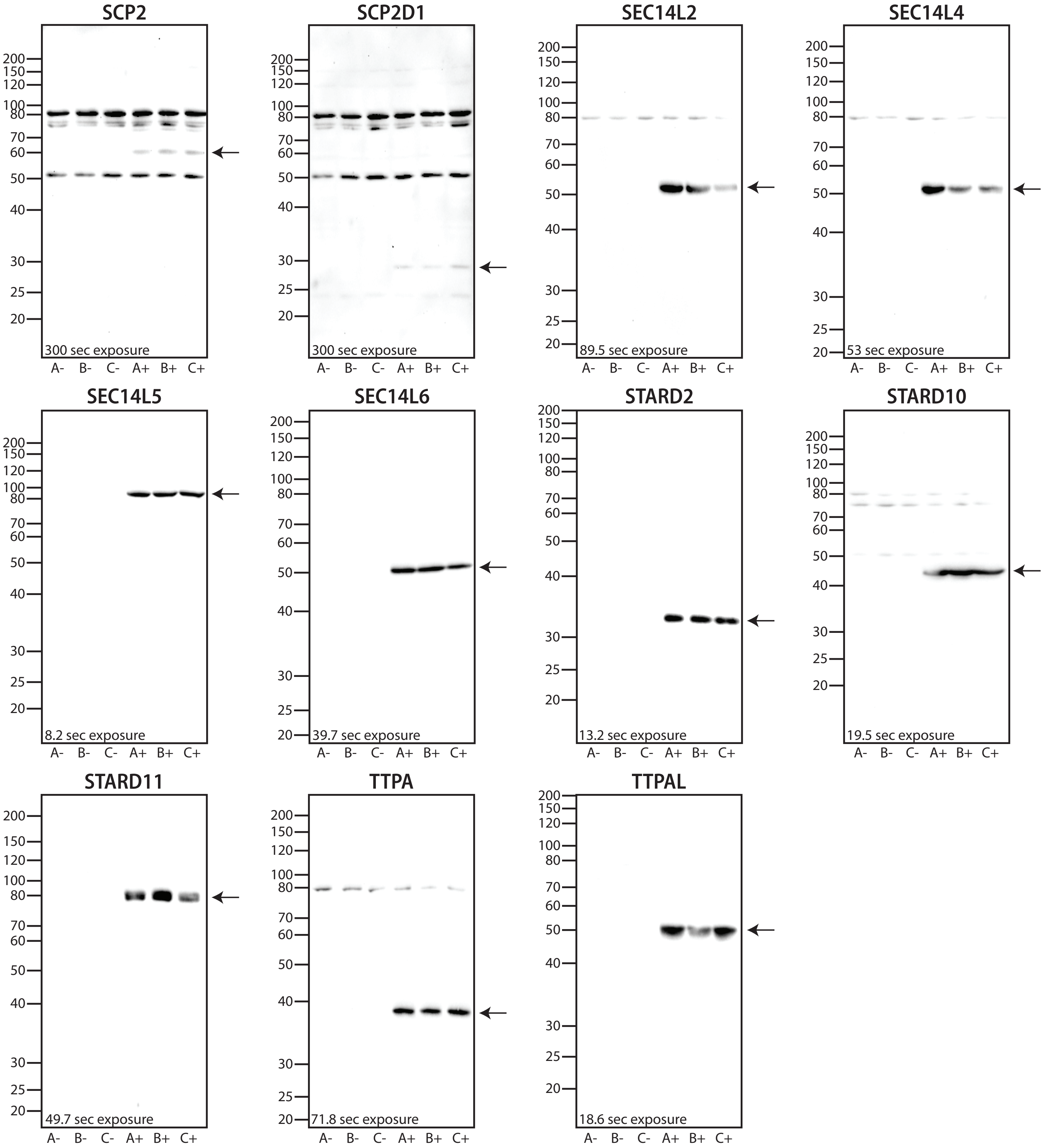
SCP2
300 sec exposure
SCP2D1
300 sec exposure
SEC14L2
89.5 sec exposure
SEC14L4
53 sec exposure
SEC14L5
8.2 sec exposure
SEC14L6
39.7 sec exposure
STARD2
13.2 sec exposure
STARD10
19.5 sec exposure
STARD11
49.7 sec exposure
TTPA
71.8 sec exposure
TTPAL
18.6 sec exposure
A- B- C- A+ B+ C+

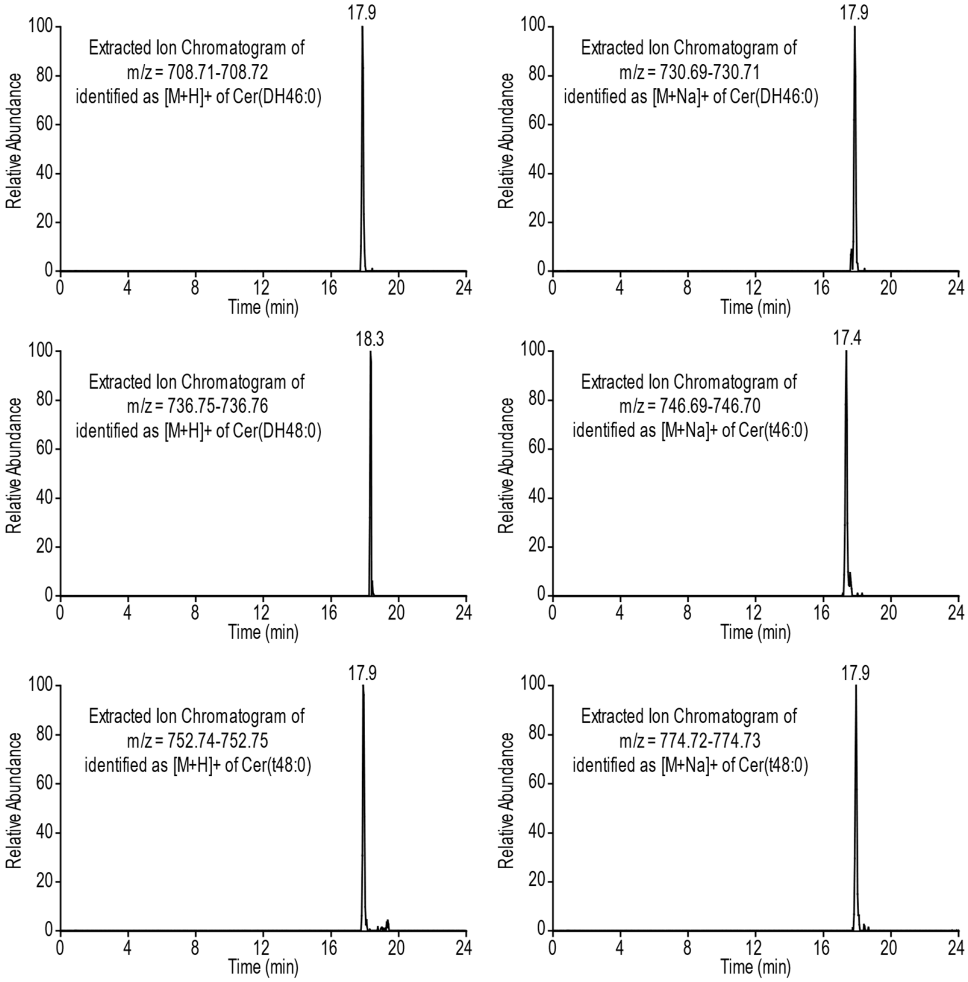

Supplementary Figure 2. EICs of unusual ceramide species eluting as different adducts. *The spectra and rules used to verify these species are in Extended Data Fig. 7b.*

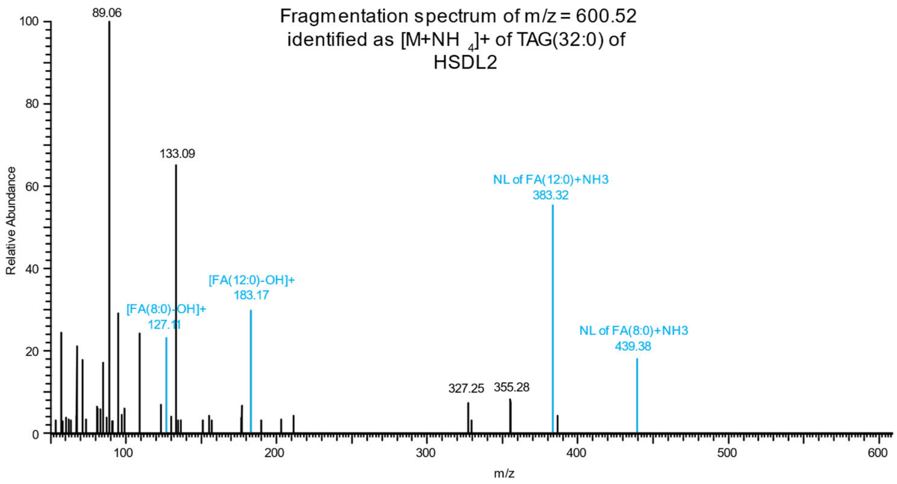

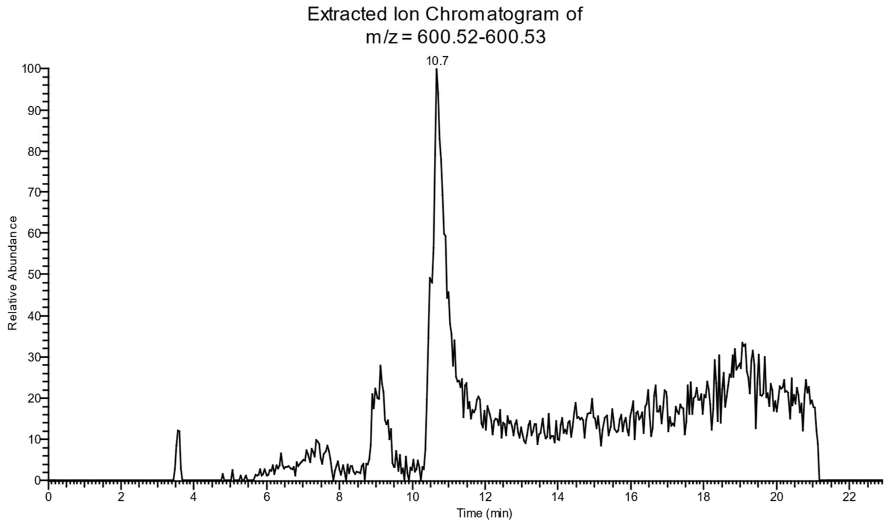

Supplementary Figure 3. Fragmentation spectra of unusual triacylglycerol species. *Upper panel: Fragmentation spectra (in blue) used to identify TAG(32:0) species. The composition of the TAG(32:0) is TAG(12:0/12:0/8:0). Lower panel: EICs of unusual TAG species.*

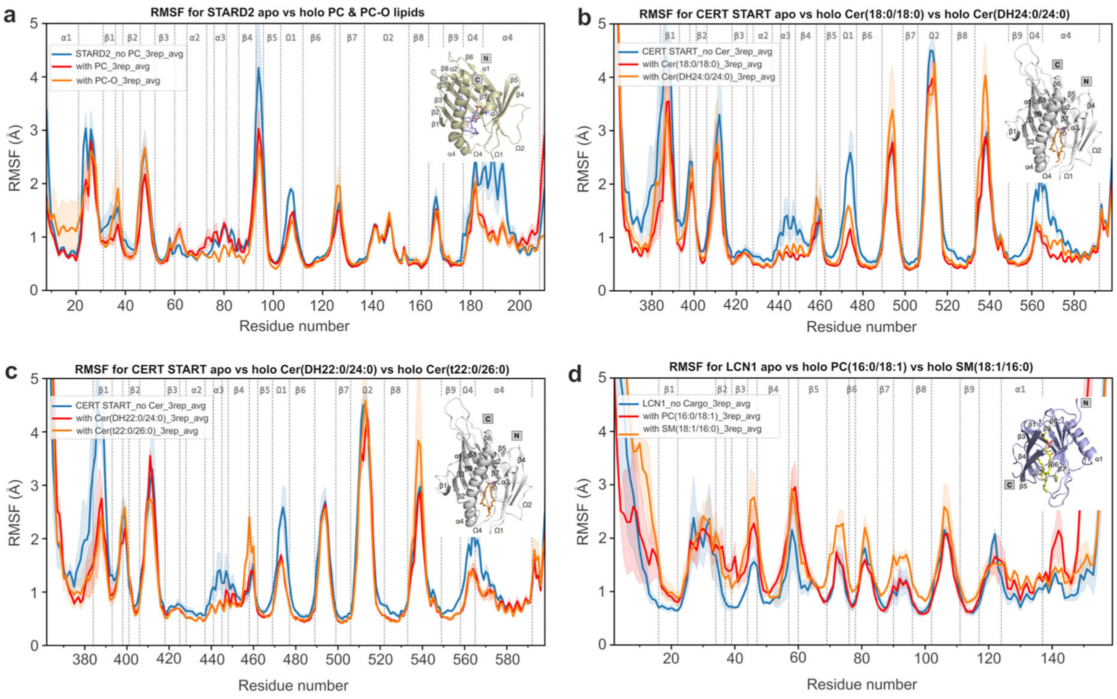

Supplementary Figure 4. Root-mean-square fluctuation (RMSF) profiles of STARD2, CERT START, and LCN1 domains in their apo and holo states with different lipid cargos. *(**a**) RMSF of STARD2 in the apo state (blue) compared with holo states bound to PC(16:0/18:2) (red) and PC-O(16:0/18:2) (orange). (**b**) RMSF of CERT START in the apo state (blue) compared with holo states bound to Cer(18:0/18:0) (red) and Cer(DH24:0/24:0) (orange). (**c**) RMSF of CERT START in the apo state (blue) compared with holo states bound to Cer(DH22:0/24:0) (red) and Cer(t22:0/26:0) (orange). (**d**) RMSF of LCN1 in the apo state (blue) compared with holo states bound to PC(16:0/18:1) (red) and SM(18:1/16:0) (orange). For each system, the RMSF values are averages over three replicate simulations, with shaded regions representing the standard error of the mean. Secondary structure elements are shown above the plots, and labelled on the holo structures displayed as insets.*

Supplementary Tables

Supplementary Table 12: Protein structures used to calculate the volume of hydrophobic pockets. For each gene name, we report the Protein Data Bank identifier of the selected structure or the Uniprot identifier[36] for the corresponding AlphaFold model[35] (gene names followed by *). We also report the values of $probe_{min}$, $probe_{max}$, B_{core} and B_{rim} and the calculated volume.

GENE	PDB/AF ID	$probe_{min}$	$probe_{max}$	B_{core}	B_{rim}	Volume (Å^3)
ATCAY*	Q86WG3	1.0	20	0.80	0.75	1906
BNIPL*	Q7Z465	1.0	20	0.80	0.75	1758
BPI	1ewf	2.0	25	0.90	0.85	2623
BPIFB2*	Q8N4F0	2.0	25	0.90	0.85	2016
CERT	6j81	2.5	10	0.87	0.80	2707
CRABP2	6hkr	1.0	30	0.90	0.80	1435
FABP1	7g00	1.0	20	0.80	0.70	1715
FABP5	7g0b	1.0	20	0.80	0.70	1506
FABP7	6l90	1.0	20	0.80	0.70	1547
GLTP	2evl	1.0	20	0.82	0.65	1802
GLTPD1	4k84	1.0	20	0.82	0.65	2005
GM2A	1g13	2.0	30	0.75	0.70	5525
HSDL2	3kvo	2.0	30	0.75	0.70	2674
LCN1	3eyc	2.0	30	0.75	0.70	2047
LCN15	2xst	1.0	30	0.65	0.60	807
OSBPL5*	Q9H0X9	2.0	30	0.92	0.85	3940
OSBPL9*	Q96SU4	2.0	30	0.92	0.85	3770
PITPNA	1uw5	1.0	20	0.82	0.73	4320
PITPNB*	P48739	1.0	20	0.85	0.75	4099
PITPNC*	Q9UKF7	1.0	20	0.85	0.75	3519
PMP2	7o60	1.0	30	0.80	0.70	1575
RBP1	8gey	1.0	30	0.75	0.65	1298
RBP4	5nub	1.0	30	0.85	0.70	1071
RBP5	6e5w	1.0	30	0.75	0.70	1548
RLBP1	4ciz	1.0	20	0.75	0.70	2195
SCP2	1qnd	1.0	20	0.90	0.80	1191
SCP2D1*	Q9UJK7	1.0	20	0.80	0.70	910
SEC14L2	4omk	2.0	5	0.92	0.85	2699
SEC14L4	4ltg	2.0	5	0.92	0.85	2402
SEC14L5*	O43304	2.5	10	0.83	0.75	1780
SEC14L6*	B5MCN3	2.0	3	0.85	0.70	2369
STARD2	7u9d	2.0	20	0.85	0.75	3492
STARD10	6ser	3.0	20	0.85	0.75	2693
TTPA	5mug	2.0	30	0.85	0.75	2261
TTPAL*	Q9BTX7	2.0	30	0.85	0.75	1907

Supplementary Table 13: Summary of the MD simulations. System compositions, simulation times, numbers of replicates and the main observations from the MD trajectories analyses.

<table>
<tr><th>Domain</th><th>Apo or ligand</th><th>Water or bilayer composition</th><th>Length (ns)</th><th>Replicates</th><th>Summary of observation</th></tr>
<tr><td rowspan="3">STARD2

UniProt ID: Q9UKL6</td><td>apo</td><td>water</td><td>500</td><td>3</td><td rowspan="3">Ether-PLPC forms stable interactions with the lipid-binding site, similar to those of a bound ester-PLPC (Extended Data Fig. 4a).

PC simulations are from Talandasthi et al, 2024 (10.1021/acs.jpclett.4c01743)</td></tr>
<tr><td>PC*</td><td>water</td><td>500</td><td>3</td></tr>
<tr><td>PLPC-O(16:0/18:2)</td><td>water</td><td>500</td><td>3</td></tr>
<tr><td rowspan="5">CERT-START

UniProt ID: Q9Y5P4</td><td>apo</td><td>water</td><td>500</td><td>3</td><td rowspan="5">Very long dihydro- or phyto-ceramides stably bound within the established ceramide-binding site (Fig. 5b & Extended Data Fig. 7d)

apo simulations are from Moqadam et al. 2024 (10.1021/acs.jpcb.4c02398)</td></tr>
<tr><td>Cer(18:0/18:0)</td><td>water</td><td>500</td><td>3</td></tr>
<tr><td>Cer(DH24:0/24:0)</td><td>water</td><td>500</td><td>3</td></tr>
<tr><td>Cer(DH22:0/24:0)</td><td>water</td><td>500</td><td>3</td></tr>
<tr><td>Cer(t22:0/26:0)</td><td>water</td><td>500</td><td>3</td></tr>
<tr><td rowspan="3">LCN1

UniProt ID: P31025</td><td>apo</td><td>water</td><td>500</td><td>3</td><td rowspan="3">The choline group of POPC or SM lipids docked into LCN1 interacts with two aromatic amino acids via cation-pi interactions that remain stable during simulations (Fig. 2c & Extended Data Fig. 5a)</td></tr>
<tr><td>POPC(16:0/18:1)</td><td>water</td><td>500</td><td>3</td></tr>
<tr><td>SM(18:1/16:0)</td><td>water</td><td>500</td><td>3</td></tr>
<tr><td>LCN1 (W17A, Y97A)</td><td>POPC(16:0/18:1)</td><td>water</td><td>500</td><td>3</td><td>POPC leaves the proposed choline headgroup binding site if the two aromatics are mutated into alanine (Extended Data Fig. 5b)</td></tr>
</table>

* Each of the three replicated simulations contains a phosphatidylcholine lipid with a different lipid tail: PLPC(16:0/18:2), DLPC(12:0/12:0), and PAPC(16:0/20:4).

Supplementary Table 14: Supplementary Tables used for the panels in the figures. NA, not applicable

FIGURE		RELATED DATA
**Figure 1**	Fig 1a	Supplementary Table 1, Supplementary Table 2
	Fig 1b	Supplementary Table 7A
	Fig 1c	Supplementary Table 7A
	Fig 1d	Supplementary Table 7C
**Figure 2**	Fig 2a	Supplementary Table 5
	Fig 2b	Supplementary Table 7C
	Fig 2c	NA
	Fig 2d	Supplementary Table 7E
**Figure 3**	Fig 3a	Supplementary Table 4
	Fig 3b	Supplementary Table 4
**Figure 4**	Fig 4a	Supplementary Table 4, Supplementary Table 10
	Fig 4b	Supplementary Table 4, Supplementary Table 7D, Supplementary Table 10
**Figure 5**	Fig 5a	Supplementary Table 4, Supplementary Table 10
	Fig 5b	NA
	Fig 5c	Supplementary Table 4, Supplementary Table 10
	Fig 5d	NA
	Ext. Data Fig. 1a	NA
	Ext. Data Fig. 1b	NA
	Ext. Data Fig. 1c	Supplementary Table 4
	Ext. Data Fig. 1d	Supplementary Table 4
	Ext. Data Fig. 2a	Supplementary Table 7A
	Ext. Data Fig. 2b	Supplementary Table 4
	Ext. Data Fig. 2c	Supplementary Table 7A
	Ext. Data Fig. 2d	Supplementary Table 7A
	Ext. Data Fig. 3	NA
	Ext. Data Fig. 4	NA
	Ext. Data Fig. 5	NA
	Ext. Data Figs. 6a	Supplementary Table 9
	Ext. Data Figs. 6b	Supplementary Table 9
	Ext. Data Figs. 6c	Supplementary Table 9
	Ext. Data Figs. 7a	Supplementary Table 11
	Ext. Data Figs. 7b	NA
	Ext. Data Figs. 7c	NA
	Ext. Data Figs. 7d	NA
	Ext. Data Fig. 8a	Supplementary Table 4, Supplementary Table 10
	Ext. Data Fig. 8b	NA

Supplementary Table 15: External datasets used (but not generated) in this work

	Name	Comment	Reference(s)	Link
Selection & cloning of human LTPs	UniProtKB		doi.org:10.1093/nar/gkt1140	www.uniprot.org/uniprotkb
Selection & cloning of human LTPs	SMART		doi: 10.1093/nar/gkj079	smart.embl.de/smart/change_mode.cgi
Selection & cloning human LTPs; definition of LTPs domains and motifs (06-2020)	InterPro		doi.org:10.1093/nar/gkaa977	www.ebi.ac.uk/interpro/
Domains and motif of LTPs (seriation)	PFAM	06/2020	doi.org:10.1093/nar/gky995	pfam.xfam.org/
Lipid identification rule-sets	SwissLipids		doi.org:10.1093/bioinformatics/btv285	swisslipids.org/
Lipid identification rule-sets	METLIN		doi.org:10.1021/acs.analchem.7b04424	metlin.scripps.edu/
Lipid identification rule-sets; SMILES for 1D lipid representations	LIPID MAPS		doi.org:10.1194/jlr.R800095-JLR200	www.lipidmaps.org/
HSDL2 interactome (Ex. Data Fig. 3b)	STRING	v. 11.0; 05/2020	doi.org:10.1093/nar/gky1131	string-db.org/
Organelle targeting motifs (Ext. Data Fig. 3c)	PSORT	05/2020	doi.org:10.1016/s0968-0004(98)01336-x	psort.org/
Organelle targeting motifs (Ext. Data Fig. 3c)	PROSITE	05/2020	doi.org:10.1093/nar/gks1067	prosite.expasy.org/
Coregulated lipids (Ext. Data Fig. 6a)	Köberlin et al.		doi.org:10.1016/j.cell.2015.05.051	
Lipid colocalisation - tissues (Ext. Data Fig. 6b)	METASPACE	All human datasets until 05-2018	doi.org:https://doi.org/10.1101/539478	metaspace2020.org/
Lipid subcellular localisation (Ext. Data Fig. 6c)	Foged et al.		doi.org:10.1101/2025.10.05.680593	
Protein Data Bank identifiers used for structures	PDB		doi.org/10.1093/nar/28.1.235	www.rcsb.org/
UniProt identifiers used for AlphaFold	UniProt	2025	doi.org:10.1093/nar/gkae1010	www.uniprot.org/
Simulated protein structures database	AlphaFold DB	2025	doi.org:10.1093/nar/gkad1011	alphafold.ebi.ac.uk/

Supplementary Methods

Selection and cloning of tagged recombinant human LTPs

We selected 125 human LTPs from literature and from protein (domain) databases (UniProtKB[52], SMART[53] and InterPro[54])[2]. Of these, 101 LTPs were cloned in frame with an N- or C-terminal His6-HA-StrepII-tag, based on the presence of a signaling peptide, in a pcDNA5/FRT/TO vector for expression in HEK293 cells (gift from the Beck laboratory, based on addgene construct 2133) (Supplementary Tables 1 and 2). Coding sequences of the full-length LTPs were obtained from commercial cDNA libraries (the Human Universal cDNA library of BioCat, Open Biosystems, Harvard Medical School), as well as from in house generated cDNA libraries. For the generation of cDNA libraries, we extracted the mRNA from HeLa, HEK293, MCF7 cells and mesenchymal stem cells using the RNeasy mini kit (Qiagen) and performed SuperScript™III RT-PCR. The primers used for the amplification of the cDNAs are described in Supplementary Table 1A. Each primer extended the two edges of the template cDNA with SfiI restrictions sites, compatible with SfiI restriction sites present on the modified pcDNA/FRT/TO plasmids in frame with the tandem affinity purification cassette. The inserted cDNAs were sequenced with both forward and reverse sequencing primers.

Using a modified version of pETM11-SUMO3 with SfiI enzyme restriction sites (provided by the EMBL Protein Expression and Purification Core Facility), an LTP-library was generated for the expression of LTPs in *E. coli*. The pETM11 vector has an N-terminal His6-TEV-SUMO3 tag and a Kanamycin resistance gene allowing antibiotic selection. The expression of genes for the pETM11 vector was under control of the Lac operon for induction of protein expression in *E.* coli. During the cloning, the cut fragments by SfiI were treated with alkaline phosphatase, and the cut inserts were separated on and purified from a 0.8% agarose gel, while the digested vectors were similarly separated from the undigested vector and possible contaminants on a 1% agarose gel. For transformation, we employed XL1-Blue Competent *E. coli*. The strains were grown overnight in Kanamycin plates at 37°C. Colony PCR was used to evaluate the presence of inserts, and the gene sequence was checked by sequencing the first 150-200 nucleotides after the first SfiI site.

Generation of the stable human cell lines (*in cellulo* approach)

HEK293 cells were maintained in DMEM, supplemented with 10% (v/v) FBS, 1% L-glutamine, and kept under selection in the presence of 15 µg/ml blasticidin and 100 µg/ml Zeocin before transfection. To create stable inducible human cell lines for LTPs, Flp-In T-Rex-293 cells (Thermofisher, Cat.Nr R78007)

or Flp-In T-REx-HeLa (Thermofisher, Cat.Nr R71407)(cells were not have been further authenticated) were co-transfected with the plasmid coding for the tagged LTP and the pOG44 plasmid encoding the Flp recombinase (Invitrogen). Positive clones were selected by adding 100 µg/ml hygromicin B and 15 µg/ml blasticidin on the day after transfection. The generation of the stable cell lines lasted each 3-4 weeks, and we generated 40 cell lines in parallel per month. In this timeframe, we expanded the cell lines in the presence of 1 µg/ml tetracycline (without other antibiotics) up to 40 p150 Thermo Scientific™ Nunc™ Cell Culture Dishes, whereafter the cells were grown further until 95% confluence (3-4 days), harvested, pelleted (yielding 2-3 ml pellet), and stored at -80°C for later use. Protein expression was evaluated by Western blot with anti-HA antibody 1:1.000 (EMBL PEPC facility; in 5% milk-TBST) (Supplementary Fig. 1), and all cell lines were tested for the presence of mycoplasma.

Protein expression and LTP-complex purifications from cell lysates (*in cellulo* approach)

Cell lysis was performed by resuspending cell pellets in lysis buffer (50 mM Tris-HCl, 250 mM NaCl, 0.5 mM DTT; 2 µM avidin, protease inhibitors cocktail (Roche) and DNAse (Roche), at pH 7.4) and leaving on ice for 20 minutes. In the case of membrane proteins and proteins closely associated to membranes, 0.5% NP40 was added to the lysis buffer to facilitate solubilization.

The final cell extract was obtained by centrifugation for 20 min at 16,000 g at 4°C in a bench top centrifuge (Eppendorf 5427R), followed by centrifugation of the previous supernatant at 49,000 rpm, using a TLA100.4 rotor. Protein-lipid complexes were isolated via the Strep-II tag, at room temperature and eluted with 5 mM biotin. The eluted complexes were centrifuged at 16,000 g for at least 5 minutes and fractionated on a Superdex 200 SEC column (Invitrogen)(Supplementary Fig. 1). After elution, protein fractions were kept separated. A part of them was used for sodium dodecyl sulfate–polyacrylamide gel electrophoresis (SDS-PAGE) on pre-cast 4-12% gradient gels (Life technologies), while the major part (100 µl) underwent lipid extraction. We performed High Performance Thin Layer Chromatography (HPTLC) in the case of the likely presence of ligands difficult to detect with mass spectrometry because of their chemical nature, such as cholesterol and cholesterol esters.

Protein expression in *E. coli* (*in vitro* approach)

The His-SUMO3 tagged proteins were expressed in BL21 Star *E. coli* cells (Invitrogen) grown in ZYM supplemented medium at 37°C until an OD600 of 1.5. Immediately after, the temperature was dropped to 18°C for the following 14h of protein overexpression. Cells were harvested at 4.000 rpm in JLA8100 rotor, resuspended and washed with PBS, centrifuged again in 50 mL falcon tubes in a

Centrifuge 5810 and snap frozen with liquid nitrogen. The *E. coli* cell pellets were stored at -80°C until the day of purification.

Liposome preparation (*in vitro* approach)

Dioleoyl phosphatidylcholine (DOPC), porcine brain polar lipid extracts and bovine liver total lipid extract were obtained from Avanti Polar Lipids. DOPC was used as a carrier lipid to help the formation of liposomes. A mixture of 1:1 molar ratio of DOPC and liver lipid extract and another mixture of 1:1 molar ratio of DOPC and brain lipid extract were prepared. Both solutions had a total amount of lipids of 3 mg. The lipid mixtures were dried into thin lipid films in glass-vials by a flow of argon gas followed by vacuum for at least 30 minutes. One millilitre of rehydration buffer (50 mM HEPES pH 7.4, 250 mM NaCl, 750 mM sucrose) was added to each lipid film in order to form liposomes to a total lipid concentration of 3 mg/ml. Vials were shaken vigorously at 62°C for at least 1h, and liposomes were subsequently downsized to 400 nm by filtering 21 times through Whatman Nuclepore Track-Etched Membranes on a mini-extruder (Avanti Polar Lipids).

Incubation of LTPs with mammalian tissue derived liposomes (*in vitro* approach)

Cell pellets were thawed on ice and resuspended in the lysis buffer (50 mM Tris pH 7.5, 250 mM NaCl, 20 mM imidazole, 0.5 mM DTT, protease inhibitors: Pefabloc 1 mM (Sigma), Bestatin 3 µM (Sigma), Aprotinin 300 nM (Sigma), E64 1 µM (Sigma), Pepstatin A 1.5 µM (Sigma), Leupeptin 1 µM (Sigma). Complete cell lysis was achieved by pressurization in Avestin Emulsiflex C3. Cell membranes and aggregated proteins were eliminated by centrifugation at 21,000 rpm for 1h in the JLA25.50 rotor. The soluble fraction was incubated with bovine liver liposomes (1:250) and porcine brain liposomes (1:250) (see next paragraph), and incubated for 1h at 4°C in in a roller mixer for falcon tubes.

Afterwards, the liposomes were separated from the lysate by centrifugation at 21,000 rpm for 1 h in the JLA25.50 rotor. Cleared lysate was incubated with Ni-NTA agarose resins (Qiagen) in order to capture the protein-lipid complexes. The affinity columns were washed with lysis buffer without protease inhibitor cocktail and cleaved overnight with TEV or SenP2 proteases. The eluted protein-complexes were loaded on a Tricorn5/150 Superdex 200 self-packed size-exclusion chromatography colum. After this, the fractions were collected to undergo the same further analysis steps as for the *in cellulo* approach.

LC-MS/MS-based measurement of LTP-associated lipids

Lipid extraction: For HPTLC analysis, lipids were extracted from samples by sequential addition of 3.75 volume chloroform:methanol (1:2 v/v), 1.25 volume chloroform, and 1.25 volume 0.5% acetic acid in 500mM NaCl followed by 30 seconds of vortexing after each step. After centrifugation at 1,200 rpm for 10 min, the bottom layer was dried under vacuum at RT in the dark and resuspended in 25 µl of a solution of chloroform:methanol (2:1).

Samples for lipid-MS analyses were prepared in the same way as described above, with a few modifications as follows. After lipid extraction, the extracted lipids were dried and resuspended in 65 µl methanol in the same glass vial. The samples were later split in two fractions and transferred into a 96-well plate (twin.tec PCR Plate 96) to be analyzed in both positive and negative ionization mode.

Lipid analysis by high-performance thin layer chromatography: Extracted and resuspended lipids were sprayed (in 25 µl) on HPTLC silica gel 60 plates (10 cm x 10 cm; Merck) in 3 mm bands using the TLC-sampler 4 (Camag). The plates were sequentially developed with a solvent system of (1) chloroform:methanol:water:acetate 65:25:4:1, and (2) cyclohexane:ethylacetate (1:1) for the analysis of polar and neutral lipids (modified from[55]). The plates were dried under vacuum and dripped in 10% (w/v) $CuSO_4$ in 8% (v/v) aqueous phosphoric acid, charred at 145°C for 4.5 min[56], and scanned for fluorescence detection using Pharos FX Plus molecular imager (Bio-Rad; 488 nm) (488 nm excitation and 530 nm emission wavelengths). Lipids applied as standards were detected with a sensitivity of <1 ng. Images were analyzed and processed using ImageJ.

Lipid analysis by liquid chromatography followed by tandem mass spectrometry: Lipids were separated on an Agilent 1260 HPLC system consisting of a degasser, binary pump, and autosampler directly coupled to a Q-Exactive Plus (Thermo), equipped with a heated ESI source. The column was a Kinetex 30 x 2.1 mm, 2.6 µm, C18, 100 Å (Phenomenex). A binary solvent system was used in order to separate lipids. The mobile phase A consisted of H_2O:acetonitrile (60:40), 10 mM ammonium formate, and 0.1% formic acid, while the mobile phase B consisted of isopropanol:acetonitrile (90:10), 10 mM ammonium formate, and 0.1% formic acid. The separation started at 80% buffer A and 20% buffer B. In a 3 min gradient, buffer B was increased from 20% to 50%, followed by a 10 min gradient from 50% to 70% buffer B. Finally, a 5.4 min gradient was applied to increase buffer B from 70% to 97%. The column was subsequently washed for 2.1 min with 97% buffer B and equilibrated for 3.6 min with 20% buffer B. The flow rate was 500 µl/min. The entire run was 24 min long. The effluent was directly introduced into the ESI source of the MS, and analyzed each time for each paired fraction in either positive or negative ionization mode. As expected from a reverse-phase LC system, lipids with long fatty acids had longer retention times than those with short fatty acids,

whereas lipids with unsaturated fatty acids eluted more rapidly than their saturated counterparts (Extended Data Fig. 1c). The ESI source ion spray voltage was set to 1.7 kV. The mass spectrometer was operated in the mass range from 250 to 1600 m/z. Charge state screening was not enabled. The ten most abundant peaks (TOP 10) were selected and fragmented in MS2 by HCD. The normalized collision energy (NCE) was 30. MS1 resolution was set to 70,000 at 200 m/z, while for the MS2 it was 17,500 at 200 m/z.

Preparation of lipid standards: We measured a series of lipid standards and determined their retention times and fragmentation patterns in order to refine the rules from the METLIN, SwissLipids and LIPID MAPS [57-59] databases to our experimental setting (Supplementary Table 3). This should ensure maximal accuracy and depth of identification at the lipid species level. This allowed to define fragmentation pattern and retention time rules for each lipid subclass. Lipid standards were dissolved in $CHCl_3MeOH$ (2:1 v/v) at concentrations ranging from 1 to 10 mg/ml and were stored at -20°C. The targeted lipid subclasses were “PC”, “PC-O”, “LPC”, “PE”, “PE-O”, “LPE”, “LPE-O”, “PS”, “PI”, “PA”, “BMP”, “PG”, “LPG”, “PGP”, “CL”, “DAG”, “TAG”, “FA”, “Vitamin A”, “Vitamin E”, “sphingosine”, “sphingosine-1-phosphate”, dCerP”, “dCer”, “DHCer”, “tCer”, “dSM”, “DHSM”, “tSM”, and the same d, DH and t subclasses for “HexCer” and the related “SHexCer” and “Hex2Cer” (Avanti Polar Lipids and Sigma).

Formulation of lipid identification rule-sets: The lipid subclass fragmentation and elution rules derived from our standards (see previous paragraph) were confirmed by spectra in the literature and public databases, such as SwissLipids, LIPID MAPS and METLIN[57-59]. The subclass rulesets are available in Supplementary Table 3, and the rules defined for the subclasses were automatically applied by the lipyd software and expanded to all theoretically possible lipid species to identify these (https://github.com/saezlab/lipyd).

Lipid identification and filtering: The raw MS data were converted to mzML format with MS Convert from the ProteoWizard package (version 3.0.679)[60]. PEAKS Studio 7.0 (Bioinformatics Solutions Inc.) was used for peak picking, feature detection and alignment. Features coming from each sample were grouped into a control group (buffer only and fractions before and after the protein peak) and a sample group (containing the fractions where the protein eluted). A first quality control accounted for the following criteria: quality (peak shape score from the PEAKS software) ≥0.2, (real sample/control) ratio >2 (performed for each protein sample fraction versus each control fraction), minimum peak intensity of 10000, only single charge states, and fitting with the retention time of lipids (above 1 minute). Finally, we considered the profile of the protein abundances over the

different SEC fractions, and only selected the potential lipid ions with an elution profile similar to the protein profile. The lipid species were computationally identified based on the above explained fragmentation rule sets. Finally, specimens that matched the protein SEC elution profile underwent a three-person manual curation that approved the final identification, using the SwissLipids database[58], also searching confirmation by taking the associated retention times into account. This manual curation also eliminated identifications that corresponded to the used detergent (NP40) in the lysis of some of the samples: samples lysed with this detergent), and we also removed any lipid identifications with ambiguity in the observed headgroups (e.g. SM vs. PC). The fact that we often identified lipid species in the form of different adducts and in different MS ionization modes also increased confidence in their identification (Extended Data Fig. 1d).

Unusual ceramide and triacylglycerol species: we identified unusual ceramide species with carbon chain lengths of 46 and 48 and triglycerides with a total carbon chain length of 32. We present the EICs in Supplementary Figure 2 and the fragmentation spectra of the ceramides with carbon lengths of 46 and 48 in comparison with ceramide standards that we measured as controls (Extended Data Fig. 7b). Supplementary Figure 3 shows the EICs, fragmentation spectra and rules used to verify the unusual TAG(32:0).

MS-intensities normalization: The MS-intensities from different screens and different lipidomics approaches were always analyzed and visualized independently, and they were normalized for representation in the (circular) heatmaps to facilitate comparisons. The intensities were first log10 transformed per mode and screen, and then min-max normalized. The min-max normalization used the minimum and maximum of the whole dataset within the particular screen and mode. These steps were followed by a scaling where maximum values correspond to 10 and minimum values to 1, while absences correspond to 0. The scaling was realized by multiplication of normalized values by 9 followed by addition of 1. After this, the results for both ion modes were compared and the maximal value was retained from either of the ion modes. The group of data under focus determines the reference frame of min-max normalization: for Fig. 2a this normalization is on the whole matrix of LTP – lipid subclass combinations (per screen independently), while for Fig. 4 this normalization is per row (per screen/lipidome independently).

For comparisons not visualized in heatmaps and where normalization is unwanted, MS-intensity sums and percentages were used, all based on linear addition of MS-intensities without transformation. Percentages that indicate the fraction of the maximal intensities (such as in the bar charts in Fig. 5a,c) show the percentage of the considered condition in comparison with the

maximum intensity of the specific row.

LC-MS/MS measurements of the effect of LTP overexpression on cellular lipidomes

Cell culture for the total lipidomics analyses: The HEK-LTP cell lines for the total lipidomics screen were selected based on whether a cargo had been found for the corresponding LTP in either the *in vitro* or *in cellulo* ligands screen.

The cell lines were thawed and placed in media (DMEM high glucose + 10% FBS + 1% P/S + 1% L-glutamine. Upon attachment of the cells to the plate, media was exchanged for media containing 15 μg/mL blasticidin and 100 μg/mL hygromycin. All cell lines were negative for mycoplasma contamination. For overexpression of the LTP, 600.000 cells/plate were seeded on 3x 10 cm dishes (biological replicates) without antibiotics and induced with 1 μg/mL tetracyclin for 72 hours (until 80-90% confluency). Cells were counted with a cell counter. Triplicates of 1.8 million cells were harvested, twice washed with PBS devoid of Mg^{2+} and Ca^{2+} and resuspended in 600 μL PBS devoid of Mg^{2+} and Ca^{2+}. The harvested cells were shipped to Lipotype for lipidomics analysis. The remainder of the harvest was kept for western blot.

Western blots: The overexpression of the LTPs was confirmed by western blot (Supplementary Fig. 1). In short, 100.000 cells/well were loaded in 4x Laemmli sample buffer (200 mM Tris pH 6.8, 8% (v/v) SDS, 40% (v/v) glycerol, 50 mM EDTA, 600 mM β-mercapto-ethanol, 0.08% (w/v) bromophenol blue). The samples were corrected with PBS to equal volumes per well. The samples and marker (PageRuler unstained protein ladder, Thermo) were run with running buffer (25 mM Tris, 192 mM glycine, 0.1% SDS) on a 10% polyacrylamide gel at 30 mA for 2 hours. The gel was equilibrated in transfer buffer (25 mM Tris, 192 mM glycine, 20% (v/v) methanol) for 30 minutes and transferred onto a nitrocellulose membrane, sandwiched between 6 buffer-soaked Wattman papers on either side, on a semi-dry transfer machine at 1 mA/cm^2 for 1 hour. The membrane was stained with ponceau staining (0.2% ponceau in 3% TCA) for 5 minutes to check the transfer efficiency and to mark the marker with a pencil. The ponceau staining was washed out with water and the membrane was blocked overnight in 5% milk-TBST (5% Tween in TBS) at room temperature. The membrane was incubated with 1:1.000 anti-HA antibody (EMBL PEPC facility; in 5% milk-TBST), 3x washed for 5 minutes with TBST, incubated with 1:10.000 anti-mouse HRP antibody (in 5% milk-TBST), washed twice with TBST and once with TBS, 1 minute incubated with ECL (West Pico Plus, Thermo) and imaged on Chemidoc.

Randomisation: During the cell culturing part of the screen, the HEK-LTP cell lines were spread across

time based on similarity of the name and size of the construct to prevent cell line swaps and to make a mistake visible in case it had happened. The original name of the construct was kept during cell culturing and only at harvesting point they were given a harvesting number for sample annotation. The sample list for Lipotype was prepared in order of harvesting number. To randomise the sample list, the Rand() function in excel was used to give a random number between 0 and 1 and sorting of the list was performed based on the given number. This randomised list was used at Lipotype as the running order of samples on the mass spectrometer.

LC-MS/MS-based lipidomics: *Lipid extraction for mass spectrometry lipidomics*: Mass spectrometry-based lipid analysis was performed by Lipotype GmbH (Dresden, Germany) as described[61]. Lipids were extracted using a two-step chloroform/methanol procedure[62]. Samples were spiked with internal lipid standard mixture containing: cardiolipin 14:0/14:0/14:0/14:0 (CL), ceramide 18:1;2/17:0 (Cer), diacylglycerol 17:0/17:0 (DAG), hexosylceramide 18:1;2/12:0 (HexCer), lyso-phosphatidate 17:0 (LPA), lyso-phosphatidylcholine 12:0 (LPC), lyso-phosphatidylethanolamine 17:1 (LPE), lyso-phosphatidylglycerol 17:1 (LPG), lyso-phosphatidylinositol 17:1 (LPI), lyso-phosphatidylserine 17:1 (LPS), phosphatidate 17:0/17:0 (PA), phosphatidylcholine 17:0/17:0 (PC), phosphatidylethanolamine 17:0/17:0 (PE), phosphatidylglycerol 17:0/17:0 (PG), phosphatidylinositol 16:0/16:0 (PI), phosphatidylserine 17:0/17:0 (PS), cholesterol ester 20:0 (CE), sphingomyelin 18:1;2/12:0;0 (SM), triacylglycerol 17:0/17:0/17:0 (TAG) and cholesterol D6 (Chol). After extraction, the organic phase was transferred to an infusion plate and dried in a speed vacuum concentrator. 1st step dry extract was re-suspended in 7.5 mM ammonium acetate in chloroform/methanol/propanol (1:2:4, V:V:V) and 2nd step dry extract in 33% ethanol solution of methylamine in chloroform/methanol (0.003:5:1; V:V:V). All liquid handling steps were performed using Hamilton Robotics STARlet robotic platform with the Anti Droplet Control feature for organic solvents pipetting.

MS data acquisition: Samples were analysed by direct infusion on a QExactive mass spectrometer (Thermo Scientific) equipped with a TriVersa NanoMate ion source (Advion Biosciences). Samples were analyzed in both positive and negative ion modes with a resolution of Rm/z=200=280000 for MS and Rm/z=200=17500 for MSMS experiments, in a single acquisition. MSMS was triggered by an inclusion list encompassing corresponding MS mass ranges scanned in 1 Da increments[63]. Both MS and MSMS data were combined to monitor CE, DAG and TAG ions as ammonium adducts; PC, PC O-, as acetate adducts; and CL, PA, PE, PE O-, PG, PI and PS as deprotonated anions. MS only was used to monitor LPA, LPE, LPE O-, LPI and LPS as deprotonated anions; Cer, HexCer, SM, LPC and LPC O- as acetate adducts and cholesterol as ammonium adduct of an acetylated derivative[64].

Data analysis and post-processing: Data were analyzed with in-house developed lipid identification software based on LipidXplorer[65,66]. Data post-processing and normalization were performed using an in-house developed data management system. Only lipid identifications with a signal-to-noise ratio >5, and a signal intensity 5-fold higher than in corresponding blank samples were considered for further data analysis.

Fluorescence emission shift binding assay

PC binding assay is adapted from (Panagabko et al.)[67] using a fluorescent analog of PC, NBD-PC. NBD-PC (810131, Avanti Polar lipids) was diluted from an ethanol stock into assay buffer (50 mM Hepes, 150 mM NaCl pH 7,4) at a final concentration of 4 µM. 1 µM of the lipid transfer domain of human CERT (CERT-START: residues 361-598), full length human STARD4 and full length human STARD10 where let to interact with NBD-PC for 30 min at room temperature. Binding was assessed as an increase of fluorescence due to insertion of the NBD-labeled fatty acid in the lipid binding pocket using Paradigm Spectramax plate reader (470 nm excitation and 543 nm emission wavelength).

Bioinformatics and statistical analyses

Protein-domain-based ordering of LTPs, and association with lipid sub-classes: The LTPs along the x-axis of Fig. 2a and along the left side of the circle of Fig. 3a were ordered by seriation based on the presence of all protein domains/motifs and their location within the proteins. The result of the underlying domain/motif-based seriation is visualized in Extended Data Fig. 3a. The protein domains/motifs and their intra-protein locations correspond to all domains and motifs that were available for these LTPs in June 2020 in either PFAM or InterPro[54,68]. The employed seriation was according to the travelling salesman algorithm, as described in[69]. In Extended Data Fig 3b, we show the results of STRING database search using version 11.0 on 05/2020[70]. For Extended Data Fig 3c, we used PSORT[71] and PROSITE[72] (on 05/2020).

Comparison of lipid co-mobilization with co-regulation and co-localization: For the validation of the observations in the screens and the discovery of biological relationships, we integrated external data from different external data sources (see further) to compare the pairs of lipids observed with the same LTPs in our data (co-mobilized lipid pairs) with their co-regulation (Extended Data Fig. 6a), and co-localization within tissues (Extended Data Fig. 6b) and within cells (Extended Data Fig. 6c) in the external data. The next paragraph describes the shared methodological and visualization elements of these analyses, while the paragraph after describes the unique aspects of each of these analyses.

In each analysis, we extracted the data from the external data source for the observed co-mobilized lipid pairs of the here-described screens and compared their distribution with the distributions of two broader sets of lipid pairs in the external data source. One broader set of lipid pairs consisted of all theoretically possible pairwise combinations of the observed lipids, instead of only the observed combinations, while the other broader set of lipids consisted of all lipid pairs in the external data, thus also including unobserved lipids. The filled coloured distributions correspond in all panels of Extended Data Fig. 6 to the results for the co-mobilized lipid pairs, while the coloured contour distributions correspond to the results for the theoretically possible combinations, and the grey contour distributions correspond to the results for all lipid pairs in the external data. The long vertical lines correspond to the medians of these distributions, respectively in black, blue/orange and grey. The calculation of the densities of these distributions used Gaussian kernels with bandwidth selection according to[73], with an overall density of 1 in all cases corresponding to the uniform distributions. Moreover, within each analysis, all distributions have the same surface area to facilitate comparisons. All analyses were performed on the aggregate of all lipid pairs ("all" in Extended Data Fig. 6), but the pairs were also split up in different subtypes of similarity to avoid domination of comparisons by closely related lipid species. The "species" subtype of lipid pairs refers to pairs that only differ in their fatty acyl chains; the "sub-class" subtype refers to pairs that differ in their linkage, for example ester-linked lipid versus ether-linked lipids or d versus DH sphingolipids; and the "class" subtype includes pairs that differ in their headgroup, for example PE and PC. The significance of the shift of the observed distribution of lipid pairs versus the external set of lipid pairs was each time determined by Fisher's exact tests[74]. The median of the expected distribution was used to split both the expected and observed data, and then the observed distribution was compared with the expected distribution. Significant results were indicated with a star in the Extended Data Fig. 6, and detailed results of the comparisons can be found in Supplementary Table 9B. The data used to study the co-regulation of co-mobilized lipids (Extended Data Fig. 6a) was derived from[6]. Co-regulations in these data represent Pearson's linear correlations in the log2-transformed changes in abundance of the lipids of the lipid pairs upon cellular perturbations. For the analysis of the co-localization in tissues (Extended Data Fig. 6b), we calculated the Manders' co-occurrence of all pairs of lipid-like molecules (as defined by ClassyFire[75]) in the mass spectrometry-based imaging metabolomics database METASPACE[35,76]. All datasets from human tissues in this database were selected up to May 2018, if there were at least 50 of the co-transported lipid species present per dataset (below 5% FDR). The background observations were removed by selecting for each dataset a metabolite with uniform distribution across the tissue, followed by morphological transformation of the corresponding image with grayscale erosion and then grayscale dilation, to define a mask representing the tissue region. We subsequently also discarded datasets where the average intensity

of the background was higher than the average intensity of the tissue region. The co-occurrences of the lipid pairs were calculated for all pixels corresponding to tissue for each of the datasets. The use of the Manders' overlap coefficient allowed to eliminate the influence on calculation of the co-localizations of the absence of both queried lipids in pixels. For analysis of the sub-cellular co-occurrence of the co-mobilized lipids (Extended Data Fig. 6c), we also employed the Manders' overlap coefficient, but on a dataset containing the lipidomes of organelles purified with antibodies (Supplementary Table 9A)[36].

Analyses of the HEK293 lipidome after overexpression of LTPs: For the analyses visualized in Fig. 1d, subsets of LTP-ligand pairs from the *in cellulo* and *in vitro* screens were mapped on and compared to all possible LTP-lipid subclass pairs in the LTP overexpression data. The fraction of matched significantly changed LTP-ligand (lipid subclasses) in each subset was each time compared to what was observed for all possible LTP-lipid subclass pairs in the LTP overexpression data, and the significance of the observed difference in these fractions was each time determined by a Fisher's exact test. The subsets "*in cellulo*" and "*in vitro*" correspond to the pairs observed respectively in the *in cellulo* and *in vitro* screens (as illustrated in more detail in Fig. 2a as the colored triangles). The subset "Both screens" corresponded to the combination of these when observed in at least one of these screens. The subsets "Novel" and "Known" have the same entries as the "*in cellulo*" and "*in vitro*" subset, but split up in a different way, namely respectively into novel or known LTP-ligand pairs (as illustrated in more detail in Fig. 2a with the white circles on the triangles (novel) or absence of these circles on the triangles (known).

The data with all possible LTP-lipid subclass pairs in the LTP overexpression data that was used for the above comparison contains information on 43 LTPs for 24 combined lipid sub-classes, corresponding to 1032 possible combinations that were either affected or not by LTP overexpression. A lipid subclass was considered affected if at least one lipid species in that lipid subclass was significantly affected by overexpression of that LTP. Supplementary Table 7C describes the results of the statistical tests for all LTP – lipid species combinations, with the significance testing performed by Welch's t-tests with Bonferroni correction for the multiple testing. The mol percentage data from Lipotype described in Supplementary Table 7B formed the basis of these statistical tests. These data underwent log10 transformation for variance stabilization, and the values of each induced sample were divided by the values of the corresponding non-induced sample. Based on this, the set of 3 sample ratios specific for the LTP under focus was compared to those of all other LTPs, to perform the significance test and the calculation of the (weighted) fold changes. The significance tests thus inherently accounted for lipid variations over all overexpression experiments, down weighting lipids

affected by multiple LTPs. The weighted fold changes were calculated by each time dividing the mean of the 3 sample ratios specific for the LTP under focus by the mean of sample ratios for the other LTPs, and these were reported and visualized after taking a log10. Fig. 2b used the same results from Supplementary Table 7C and directly visualized the lipid species changes for each of the LTPs under focus, and the figure also included colored highlights for the lipid sub-classes of interest for focused description.

The results in the "perturbed lipidome" heatmaps at the bottom of Fig. 4b were ultimately also based on the Supplementary Table 7B. For each set of samples in this table that corresponds to the LTP under focus, a paired t-test was performed comparing each set of 3 induced samples with the 3 corresponding non-induced samples at species level for all glycerophospholipids, and the results of these tests were included in Supplementary Table 7D. These species level results were then binned based on their fatty acids characteristics in focus, respectively for both heatmaps either the different total chainlengths or unsaturations in the relevant range for the comparison with the "mobilized lipidome" in the figure. Hereafter, the fraction of significantly changed species versus non-significantly changed species was determined per bin, and the results were min-max normalized over all bins for the particular fatty acids characteristics in focus, respectively total carbon chain length or total unsaturation.

Data analysis scripts availability and programming context: The in-house developed library for lipid identification and matching of lipid elution profiles with protein elution profile is called Lipyd and is accessible via https://github.com/saezlab/lipyd. The R scripts for the data clean-up, and data analyses and visualizations are available via https://github.com/krtiteca/ScriptsAssociatedWithLTPArticle. For these analyses and visualization, we consequently used R version 3.5.0. For the visualization of beanplots, we employed the R package by[77], and for the circular visualization and heatmaps, we employed the packages circlize and ComplexHeatmap[78,79].

Analysis of LTP and lipid volumes

Calculation of lipid volumes: To compute the volume of each lipid we leveraged a simple approach for fast approximation of the volume of small molecules based on the atomic van der Waals radii ri[80,81]:

$$V_{vdw} = \sum_{i=1}^{L} \frac{4}{3}\pi r_i^3 - 5.92N_B - 14.7R_A - 3.8\,R_{NA}$$

where N_B is the number of bonds, L the number of atoms, R_A the number of aromatic rings, and R_{NA} the number of nonaromatic rings. R_A and R_{NA} are null for the lipids considered. The number and nature of atoms and bonds was calculated with the RDKit toolset (Open-source cheminformatics. https://www.rdkit.org) from a one-dimensional representation of the lipids (Simplified Molecular Input Line Entry System, SMILES). The SMILES were obtained with the LipidMaps database[82] and for all the lipid head groups and chain fragments present in the mass spectrometry data.

Detection of LTP pockets and calculation of their volumes with Voronota: To identify the protein pockets and compute their volumes we used Voronota[25], a software tool for analyses of biomolecular structures using Voronoi tessellation. In the context of a protein structural model, a pocket is defined as a region of unoccupied space that is at least partially enclosed by protein atoms. We employed the voronota-pocket script described further down, and available at https://github.com/kliment-olechnovic/voronota/blob/master/voronota-pocket.

Calculating and filtering tangent spheres that fill the empty space. Given a protein structure represented as a set of atomic balls of van der Waals radii, Voronota calculates geometric entities called Voronoi vertices. Each Voronoi vertex inherently defines a tangent sphere that touches exactly four atomic balls and does not intersect any other atomic balls. Such tangent spheres are utilized to describe empty space both inside and outside the protein. The tangent spheres are filtered based on their radii (minimum and maximum radius thresholds, respectively called $probe_{min}$ and $probe_{max}$, are adjustable parameters).

Estimating buriedness of a tangent sphere. To determine whether a tangent sphere is buried within the protein, neighboring atoms of the sphere are analyzed. The neighbors consist of the four atoms touched by the sphere and additional atoms located within a defined proximity threshold (an adjustable parameter). For each neighboring atomic ball, the nearest point on the surface of the tangent sphere, termed the neighbor point, is calculated. Then two values, S and S_{max}, are computed: S is the total sum of pairwise distances between neighbor points on the tangent sphere; S_{max} is the maximum possible total sum of distances for the same number of points arranged on a circle with radius equal to that of the tangent sphere. It was proven by Toth that the sum of the pairwise distances determined by N points on a circle is maximized when the points are the vertices of a regular N-sided polygon inscribed in the circle[83]. Thus, for N points and radius r, $S_{max} = (rN)/\tan\left(\pi/(2N)\right)$. S approaches or exceeds S_{max} only if the tangent sphere is surrounded by protein atoms from multiple directions, indicating burial within the protein structure. Conversely, S approaches zero if neighboring atoms concentrate within a narrow region, failing to surround the

sphere fully or even partially. From these considerations, the buriedness value B for a tangent sphere is defined as $B = \min(S/S_{max}, 1)$. Thus, B approaches 1 for spheres deeply buried inside the protein and approaches 0 for spheres located near the protein's outer surface.

Calculating buriedness of atoms from the buriedness of tangent spheres. For each atom, the buriedness value is calculated as a weighted average of the buriedness values of its contacting tangent spheres, with the volumes of these tangent spheres serving as weights. Subsequently, a graph of atoms is constructed, wherein each node corresponds to an atom associated with a buriedness value, and edges connect pairs of atoms sharing at least one tangent sphere. This graph is initially utilized to smooth and denoise the buriedness values: each atom's buriedness is replaced by the average of its own and neighboring atoms' buriedness values in the graph.

Identifying sets of pocket atoms. The graph constructed previously is employed to identify connected subgraphs corresponding to sets of atoms surrounding pockets. The subgraph construction commences by selecting an atom whose buriedness value exceeds the provided B_{core} threshold. Then, neighboring atoms whose buriedness values surpass the B_{rim} thresholds are progressively added to the subgraph. Construction of each subgraph terminates once no further atoms satisfying the threshold conditions can be included. Additional subgraphs are iteratively constructed until no atoms with buriedness values above the B_{core} threshold remain unassigned. The largest resulting subgraph is selected, and its nodes define the set P of atoms surrounding the largest pocket.

Calculating the volume of the pocket. For the set P of pocket atoms identified previously, tangent spheres touching exclusively atoms from P are selected. As each tangent sphere contacts exactly four atoms, the centers of these atoms naturally form a tetrahedron. Because tangent spheres directly correspond to Voronoi vertices, the constructed tetrahedra do not overlap, permitting their individual volumes to be summed directly. The estimated total pocket volume is calculated as the sum of the volumes of all tetrahedra corresponding to the selected tangent spheres.

Running the Voronota software. We calculated the volumes of the pockets in each of the structure listed in Table 12 (below). Note that we used experimentally determined protein structures whenever available (see PDB ID in Table 12 below), but also structures from the AlphaFold database[24], of which we extracted the annotated LTP domain. The pocket definition and volume calculation are executed with a single command calling the voronota-pocket script:

```
voronota-pocket -i {PDB} --probe-min 1 --probe-max 30 --buriedness-core 0.8 --buriedness-rim 0.7 --subpockets 1
```

where--probe-min, --probe-max, --buriedness-core and --buriedness-rim serve to input the respective values of $probe_{min}$, $probe_{max}$, B_{core} and B_{rim} defined above.

The script does not input ligands, ensuring that there are no occupied pockets in the analyzed structure. Each structure was visually inspected with PyMol to verify that the hydrophobic pockets were correctly identified by Voronota. Consequently, the input parameters were adjusted for each protein (Supplementary Table 12).

System preparation and MD simulation protocols

In order to provide structural models of LTPs bound to novel lipid cargos identified *in vitro* and *in cellulo*, we performed a series of molecular dynamics (MD) simulations at the atomistic level of resolution. The analyses focused on the identification and characterization of the strength of enthalpically relevant LTP-cargo interactions, such as hydrogen bonds, cation-pi-interactions or hydrophobic contacts. The simulations are listed in Table 13 below and the details of the computational protocols are described in the following paragraphs. All simulations were performed using the NAMD3 simulation package[84] and the CHARMM36m force field[85-87], with its extension for WYF-choline cation-π interactions[88,89]. All simulations were triplicated using a different distribution of velocities for each replica (Supplementary Table 13).

START-Dihydroceramide and START-Phytoceramide: We retrieved X-ray structures of CERT START domain in complex with ceramide from the Protein Data Bank (PDB)[90] (PDB ID: 2E3Q)[91] as our reference structure. To construct the complex of the START domain with dihydroceramide molecules, the ceramide molecule in the reference complex was modified to represent dihydroceramide by converting the double bond in the sphingosine chain to a single bond. Then the hydrocarbon tails of the dihydroceramide were extended to construct two variants of the complex: Cer(DH24:0/24:0) and Cer(DH22:0/24:0). To build the complex of the START domain with phytoceramide molecules, we replaced the ceramide molecule in the reference structure with a tCeramide molecule using CHARMM-GUI[92,93] and its CHARMM Small Molecule Library. Then the hydrocarbon tails of the phytoceramide were modified to construct the START domain in complex with Cer(t22:0/26:0). All the modifications were performed using PyMol[94]. To ensure structural alignment, each molecule was built to align closely with the ceramide structure in the reference START-ceramide complex ensuring minimal disruption to interactions with the START domain. The molecule parameters were generated using CHARMM General Force Field (CGenFF) program version 2.5.1 through the CHARMM-GUI server. Then, a single-point energy calculation was performed using the CHARMM-GUI PDB Reader & Manipulator[95]. This step ensured that the atomic coordinates were successfully defined and that the

structure was ready to use in the CHARMM-GUI Solution Builder tool[4]. Each complex was solvated with TIP3P water model[96] using CHARMM-GUI in a periodic cubic box (dimension: 89 Å). Potassium ions were used to neutralize the simulated systems. The systems were first subjected to energy minimization with conjugate gradients (10,000 steps) and followed by 10 ns NVT equilibrations. Position restraints using a harmonic spring potential were applied to all atoms of the ceramide molecules except hydrogens. Next, the production run for each system was performed for 500 ns using the coordinates and velocities of the last step of the equilibration run. The production run was performed with an integration step of 2 fs in the NPT ensemble. The temperature and pressure were set at 310 K and 1 bar, respectively. Langevin dynamics with a temperature damping coefficient of 1.0 and the Langevin piston method with an oscillation period of 50 fs and a damping timescale of 25 fs were used to control the temperature and pressure, respectively. The ratio of the unit cell in the x–y plane was kept constant. Electrostatic potentials were calculated using the particle mesh Ewald (PME) method[97]. A Lennard-Jones switching function of 10–12 Å was used for van der Waals interactions. Graphical representations were rendered using PyMol (version 3.0.0)[5] and VMD (version 1.9.3)[98].

LCN1-POPC(16:0/18:1) and LCN1-spingomyelin (SM(18:1/16:0): We retrieved the NMR Structure of the apo form of the human tear lipocalin (LCN1) from the protein databank (PDB ID: 5T43). To build the structure of LCN1 bound to phosphatidylcholine lipid, we docked a POPC lipid inside the pocket using the EDock webserver[99]. The obtained LCN1-POPC complex was prepared for simulation as described above for CERT. The complex was carefully optimized through multiple equilibration steps in the NVT ensemble with decreasing restraints on the atoms others than hydrogens to assure smooth relaxation of the complex. Finally, we performed 500 ns simulation in the NPT ensemble without any restraints.

Using the structure of the LCN1-POPC complex, the POPC molecule was replaced by a sphingomyelin (SM(18:1/16:0)) molecule. The SM structure was obtained from the CHARMM-GUI Individual Lipid Molecule Library[100]. The phosphate and choline groups of SM were aligned to the corresponding groups in POPC to dock the head group in the lipid binding site. The resulting LCN1-SM complex was prepared for simulation by performing energy minimization using the CHARMM program[101] (version 49a2). The complex was solvated with water molecules and neutralized with sodium ions. The simulations were performed with the same protocol as for START-dihydroceramide (and -phytoceramide). The production run was performed for 500 ns.

Apo STARD2: As no experimental structure of apo STARD2 is available, we generated a model by

removing the PLPC(16:0/18:2) lipid from the holo STARD2 structure (PDB ID: 1LN3)[34]. After minimization (10,000 CG steps), the protein was carefully equilibrated (10 ns NVT; four 5 ns NPT steps with decreasing restraints of 10, 5, 2.5, 1, and 0.5 kcal/mol), followed by a 500 ns unrestrained NPT simulation in water.

***STARD2-PLPC(16:0/18:2) and STARD2-PLPC-O(16:0/18:2)*:** The X-ray structure of STARD2 in complex with PLPC was taken from the PDB (ID: 1LN3)[34]. To construct the complex of STARD2 with ether PLPC (PLPC-O) we used the complex from RCSB and modified the lipid using PyMol to create PLPC-O. Then, the topology and parameters were generated using CGenFF version 2.5.1 through CHARMM-GUI. The system preparation and simulation parameters were the same as for the simulations of CERT, except that we employed hydrogen mass repartitioning[102,103] (HMR) to reduce the computational costs.

MD trajectory analyses

To analyze the MD trajectories, we used Charmm[101] and an in-house code based on the MDAnalysis package[104]. Criteria for detection of hydrogen bonds and cation-π interactions were as reported earlier[13,31]: 2.4 Å or less for the acceptor-hydrogen distance and 130 degrees for the acceptor-hydrogen-donor angle, a criteria for cation- π interactions of maximum 7 Å for all distances between the aromatic carbon and choline nitrogen engaged. The position of the lipid within the LCN1 binding pocket was also assessed by measuring the distances between the nitrogen atom of the choline moiety and the β-carbons of residues 17 and 97 in wild-type and mutant LCN1 using VMD. Root mean square fluctuations (RMSF) of Cα atoms were calculated in VMD over the final 200 ns of the production trajectories. All frames were aligned to the protein backbone, and fluctuations were measured relative to the average position of each Cα atom.

The RMSF profiles of CERT, STARD2 and LCN1 are shown in Supplementary Figure 4 and are characteristic of stable protein-ligand complexes. The known ligands of CERT and STARD2 (ceramide and phosphatidylcholine, respectively) caused a slight decrease in RMSF, particularly noticeable in the gate region (Ω1 and Ω4α4). The new ligands, ether phosphatidylcholine and long DH-ceramide, respectively, had similar effects as the known ones. In the case of LCN1, the known, PC, and novel, SM, cargos also had similar effects, but they tend to increase the RMSF of LCN1 in the gate region. This is due to the high flexibility of several of the LCN1 loops which are long and unstructured (eg. β1β2, β5β6 and β6β7, β8β9). This high flexibility is corroborated by (1) the large standard error in the RMSF calculated for the three replicates of the *apo* simulations and (2) the large structural difference that exists between the 20 conformations resolved by NMR (PDB ID: 5T43) between which the backbone root mean square deviation reaches up to 2 Å.

Data availability statement

The lipidomics data can be downloaded from https://www.ebi.ac.uk/metabolights/MTBLS9567. Organelle lipidomics data are available at https://doi.org/10.1101/2025.10.05.680593.

The source data are organized as followed: molecular biology and LTP expression, MS fragmentation behaviour Supplementary Tables 1,2 and 3, respectively; lipid species or subclasses bound to LTP, Supplementary Tables 4 and 5, respectively; LTPs lipidated in only one of the assays, Supplementary Table 6; Results of the structural and functional benchmarks, Supplementary Tables 7 and 8; Functional relationship of lipids co-mobilized by the same LTP, Supplementary Table 9; Lipidomic data of bovine liver / brain extracts and HEK293 cells, Supplementary Table 10; Lipidomics CERT-over-expressing HeLa cells, Supplementary Table 11. Supplementary Tables used to produce the panels of the figures are summarized in Supplementary Table 14. Gels, SEC profiles and western blots are provided in Supplementary Figure 1.

External datasets analysed (but not generated) in this work are: sequences of human LTPs, UniProtKB (https://www.uniprot.org/uniprotkb) and SMART (https://smart.embl.de/smart/change_mode.cgi); definition of domains and motifs, InterPro (https://www.ebi.ac.uk/interpro/) and PFAM (http://pfam.xfam.org/); lipid identification rule-sets, SwissLipids (https://swisslipids.org/) and METLIN (https://metlin.scripps.edu/); SMILES for 1D lipid representations, LIPID MAPS (https://www.lipidmaps.org/); analyses of HSDL2 protein interactome, STRING (https://string-db.org/); analyses of organelle targeting sequences, PSORT (https://psort.org/) and PROSITE (https://prosite.expasy.org/); coregulated lipids, https://doi.org:10.1016/j.cell.2015.05.051; lipid colocalization, METASPACE (https://metaspace2020.org/); lipid subcellular localisation, https://doi.org:10.1101/2025.10.05.680593; protein structures, PDB (https://www.rcsb.org/); simulated protein structures, AlphaFold DB (https://alphafold.ebi.ac.uk/). External datasets analysed (but not generated) in this work are summarized in Supplementary Table 15.

References

52 UniProtConsortium. Activities at the Universal Protein Resource (UniProt). *Nucleic Acids Res* **42**, D191-198 (2014). https://doi.org:10.1093/nar/gkt1140.

53 Letunic, I. *et al.* SMART 5: domains in the context of genomes and networks. *Nucleic Acids Res* **34**, D257-260 (2006).

54 Blum, M. *et al.* The InterPro protein families and domains database: 20 years on. *Nucleic Acids Res* **49**, D344-D354 (2021). https://doi.org:10.1093/nar/gkaa977

55 Weerheim, A. M., Kolb, A. M., Sturk, A. & Nieuwland, R. Phospholipid composition of cell-derived microparticles determined by one-dimensional high-performance thin-layer chromatography. *Anal Biochem* **302**, 191-198 (2002). https://doi.org:10.1006/abio.2001.5552

56 Churchward, M. A., Brandman, D. M., Rogasevskaia, T. & Coorssen, J. R. Copper (II) sulfate charring for high sensitivity on-plate fluorescent detection of lipids and sterols: quantitative analyses of the composition of functional secretory vesicles. *J Chem Biol* **1**, 79-87 (2008). https://doi.org:10.1007/s12154-008-0007-1

57 Guijas, C. *et al.* METLIN: A Technology Platform for Identifying Knowns and Unknowns. *Anal Chem* **90**, 3156-3164 (2018). https://doi.org:10.1021/acs.analchem.7b04424

58 Aimo, L. *et al.* The SwissLipids knowledgebase for lipid biology. *Bioinformatics* **31**, 2860-2866 (2015). https://doi.org:10.1093/bioinformatics/btv285

59 Fahy, E. *et al.* Update of the LIPID MAPS comprehensive classification system for lipids. *J Lipid Res* **50 Suppl**, S9-14 (2009). https://doi.org:10.1194/jlr.R800095-JLR200

60 Chambers, M. C. *et al.* A cross-platform toolkit for mass spectrometry and proteomics. *Nat Biotechnol* **30**, 918-920 (2012). https://doi.org:10.1038/nbt.2377

61 Sampaio, J. L. *et al.* Membrane lipidome of an epithelial cell line. *Proc Natl Acad Sci U S A* **108**, 1903-1907 (2011). https://doi.org:10.1073/pnas.1019267108

62 Ejsing, C. S. *et al.* Global analysis of the yeast lipidome by quantitative shotgun mass spectrometry. *Proc Natl Acad Sci U S A* **106**, 2136-2141 (2009). https://doi.org:10.1073/pnas.0811700106

63 Surma, M. A. *et al.* An automated shotgun lipidomics platform for high throughput, comprehensive, and quantitative analysis of blood plasma intact lipids. *Eur J Lipid Sci Technol* **117**, 1540-1549 (2015). https://doi.org:10.1002/ejlt.201500145

64 Liebisch, G. *et al.* High throughput quantification of cholesterol and cholesteryl ester by electrospray ionization tandem mass spectrometry (ESI-MS/MS). *Biochim Biophys Acta* **1761**, 121-128 (2006). https://doi.org:10.1016/j.bbalip.2005.12.007

65 Herzog, R. *et al.* A novel informatics concept for high-throughput shotgun lipidomics based on the molecular fragmentation query language. *Genome Biol* **12**, R8 (2011). https://doi.org:10.1186/gb-2011-12-1-r8

66 Herzog, R. *et al.* LipidXplorer: a software for consensual cross-platform lipidomics. *PLoS One* **7**, e29851 (2012). https://doi.org:10.1371/journal.pone.0029851

67 Panagabko, C., Baptist, M. & Atkinson, J. In vitro lipid transfer assays of phosphatidylinositol transfer proteins provide insight into the in vivo mechanism of ligand transfer. *Biochim Biophys Acta Biomembr* **1861**, 619-630 (2019). https://doi.org:10.1016/j.bbamem.2018.12.003

68 El-Gebali, S. *et al.* The Pfam protein families database in 2019. *Nucleic Acids Res* **47**, D427-D432 (2019). https://doi.org:10.1093/nar/gky995

69 Hahsler, M., Hornik, K. & Buchta, C. Getting Things in Order: An Introduction to the R Package seriation. *Journal of Statistical Software* **25**, 1 - 34 (2008). https://doi.org:10.18637/jss.v025.i03

70 Szklarczyk, D. *et al.* STRING v11: protein-protein association networks with increased coverage, supporting functional discovery in genome-wide experimental datasets. *Nucleic Acids Res* **47**, D607-D613 (2019). https://doi.org:10.1093/nar/gky1131
71 Nakai, K. & Horton, P. PSORT: a program for detecting sorting signals in proteins and predicting their subcellular localization. *Trends Biochem Sci* **24**, 34-36 (1999). https://doi.org:10.1016/s0968-0004(98)01336-x
72 Sigrist, C. J. *et al.* New and continuing developments at PROSITE. *Nucleic Acids Res* **41**, D344-347 (2013). https://doi.org:10.1093/nar/gks1067
73 Sheather, S. J. & Jones, M. C. A Reliable Data-Based Bandwidth Selection Method for Kernel Density Estimation. *Journal of the Royal Statistical Society: Series B (Methodological)* **53**, 683-690 (1991). https://doi.org:https://doi.org/10.1111/j.2517-6161.1991.tb01857.x
74 Fisher, R. A. *Statistical methods for research workers, 5th ed*. (Edinburgh, 1934).
75 Djoumbou Feunang, Y. *et al.* ClassyFire: automated chemical classification with a comprehensive, computable taxonomy. *J Cheminform* **8**, 61 (2016). https://doi.org:10.1186/s13321-016-0174-y
76 Manders, E. M. M., Verbeek, F. J. & Aten, J. A. Measurement of co-localization of objects in dual-colour confocal images. *J Microsc* **169**, 375-382 (1993). https://doi.org:10.1111/j.1365-2818.1993.tb03313.x
77 Kampstra, P. Beanplot: A Boxplot Alternative for Visual Comparison of Distributions. *Journal of Statistical Software, Code Snippets* **28**, 1 - 9 (2008). https://doi.org:10.18637/jss.v028.c01
78 Gu, Z., Gu, L., Eils, R., Schlesner, M. & Brors, B. circlize Implements and enhances circular visualization in R. *Bioinformatics* **30**, 2811-2812 (2014). https://doi.org:10.1093/bioinformatics/btu393
79 Gu, Z., Eils, R. & Schlesner, M. Complex heatmaps reveal patterns and correlations in multidimensional genomic data. *Bioinformatics* **32**, 2847-2849 (2016). https://doi.org:10.1093/bioinformatics/btw313
80 Zhao, Y. H., Abraham, M. H. & Zissimos, A. M. Fast calculation of van der Waals volume as a sum of atomic and bond contributions and its application to drug compounds. *J Org Chem* **68**, 7368-7373 (2003). https://doi.org:10.1021/jo034808o
81 Bondi, A. Van der Waals volumes and radii. *The Journal of Physical Chemistry* **68**, 441 (1964).
82 Conroy, M. J. *et al.* LIPID MAPS: update to databases and tools for the lipidomics community. *Nucleic Acids Res* **52**, D1677-D1682 (2024). https://doi.org:10.1093/nar/gkad896
83 Tóth, L. F. On the sum of distances determined by a pointset. *Acta Mathematica Academiae Scientiarum Hungarica* **7**, 397-401 (1956). https://doi.org:10.1007/BF02020534
84 Phillips, J. C. *et al.* Scalable molecular dynamics on CPU and GPU architectures with NAMD. *The Journal of Chemical Physics* **153**, 044130 (2020). https://doi.org:10.1063/5.0014475
85 Venable, Richard M. *et al.* CHARMM All-Atom Additive Force Field for Sphingomyelin: Elucidation of Hydrogen Bonding and of Positive Curvature. *Biophysical Journal* **107**, 134-145 (2014). https://doi.org:10.1016/j.bpj.2014.05.034
86 Best, R. B. *et al.* Optimization of the additive CHARMM all-atom protein force field targeting improved sampling of the backbone φ, ψ and side-chain χ1 and χ2 dihedral angles. *Journal of Chemical Theory and Computation* **8**, 3257-3273 (2012). https://doi.org:10.1021/ct300400x
87 Klauda, J. B. *et al.* Update of the CHARMM All-Atom Additive Force Field for Lipids: Validation on Six Lipid Types. *Journal of Physical Chemistry B* **114**, 7830-7843 (2010). https://doi.org:10.1021/jp101759q
88 Khan, H. M. *et al.* Improving the Force Field Description of Tyrosine-Choline Cation-pi Interactions: QM Investigation of Phenol-N(Me)(4)(+) Interactions. *J Chem Theory Comput* **12**, 5585-5595 (2016). https://doi.org:10.1021/acs.jctc.6b00654
89 Khan, H. M., MacKerell, A. D., Jr. & Reuter, N. Cation-pi Interactions between Methylated Ammonium Groups and Tryptophan in the CHARMM36 Additive Force Field. *J Chem Theory Comput* **15**, 7-12 (2019). https://doi.org:10.1021/acs.jctc.8b00839

90 Berman, H. M. *et al.* The Protein Data Bank. *Nucleic Acids Res* **28**, 235-242 (2000). https://doi.org/10.1093/nar/28.1.235.

91 Kudo, N. *et al.* Structural basis for specific lipid recognition by CERT responsible for nonvesicular trafficking of ceramide. *Proceedings of the National Academy of Sciences* **105**, 488-493 (2008). https://doi.org:10.1073/pnas.0709191105

92 Jo, S., Kim, T., Iyer, V. G. & Im, W. CHARMM-GUI: a web-based graphical user interface for CHARMM. *J Comput Chem* **29**, 1859-1865 (2008). https://doi.org:10.1002/jcc.20945

93 Lee, J. *et al.* CHARMM-GUI Input Generator for NAMD, GROMACS, AMBER, OpenMM, and CHARMM/OpenMM Simulations Using the CHARMM36 Additive Force Field. *J Chem Theory Comput* **12**, 405-413 (2016). https://doi.org:10.1021/acs.jctc.5b00935

94 Schrödinger, L. (2015).

95 Jo, S. *et al.* CHARMM-GUI PDB manipulator for advanced modeling and simulations of proteins containing nonstandard residues. *Adv Protein Chem Struct Biol* **96**, 235-265 (2014). https://doi.org:10.1016/bs.apcsb.2014.06.002

96 Jorgensen, W. L., Chandrasekhar, J., Madura, J. D., Impey, R. W. & Klein, M. L. Comparison of simple potential functions for simulating liquid water. *The Journal of Chemical Physics* **79**, 926-935 (1983). https://doi.org:10.1063/1.445869

97 Essmann, U. *et al.* A smooth particle mesh Ewald method. *The Journal of Chemical Physics* **103**, 8577-8593 (1995). https://doi.org:10.1063/1.470117

98 Humphrey, W., Dalke, A. & Schulten, K. VMD: Visual molecular dynamics. *Journal of Molecular Graphics* **14**, 33-38 (1996). https://doi.org:10.1016/0263-7855(96)00018-5

99 Zhang, W., Bell, E. W., Yin, M. & Zhang, Y. EDock: blind protein–ligand docking by replica-exchange monte carlo simulation. *Journal of Cheminformatics* **12**, 37 (2020). https://doi.org:10.1186/s13321-020-00440-9

100 Jo, S., Kim, T. & Im, W. Automated builder and database of protein/membrane complexes for molecular dynamics simulations. *PLOS ONE* **2**, e880 (2007). https://doi.org:10.1371/journal.pone.0000880

101 Brooks, B. R. *et al.* CHARMM: The biomolecular simulation program. *Journal of Computational Chemistry* **30**, 1545-1614 (2009). https://doi.org:10.1002/jcc.21287

102 Gao, Y. *et al.* CHARMM-GUI Supports Hydrogen Mass Repartitioning and Different Protonation States of Phosphates in Lipopolysaccharides. *Journal of Chemical Information and Modeling* **61**, 831-839 (2021). https://doi.org:10.1021/acs.jcim.0c01360

103 Balusek, C. *et al.* Accelerating Membrane Simulations with Hydrogen Mass Repartitioning. *Journal of Chemical Theory and Computation* **15**, 4673-4686 (2019). https://doi.org:10.1021/acs.jctc.9b00160

104 Gowers, R. J. *et al.* MDAnalysis: A Python Package for the Rapid Analysis of Molecular Dynamics Simulations. *scipy* (2016). https://doi.org:10.25080/Majora-629e541a-00e
