# Supplementary material for: Systematic analyses of lipid mobilization by human lipid transfer proteins
Source: Nature. 2026 Jan 7;651(8105):511–20. doi: 10.1038/s41586-025-10040-y (PMC12979188; doi:10.1038/s41586-025-10040-y)
Supplement: Supplementary file 2 — Reporting Summary [file 41586_2025_10040_MOESM2_ESM.pdf]

nature portfolio

Corresponding author(s): Anne-Claude Gavin and Maro Hennrich

Last updated by author(s): Dec 9, 2025

Reporting Summary

Nature Portfolio wishes to improve the reproducibility of the work that we publish. This form provides structure for consistency and transparency in reporting. For further information on Nature Portfolio policies, see our Editorial Policies and the Editorial Policy Checklist.

Statistics

For all statistical analyses, confirm that the following items are present in the figure legend, table legend, main text, or Methods section.

n/a	Confirmed	
☐	☒	The exact sample size (*n*) for each experimental group/condition, given as a discrete number and unit of measurement
☐	☒	A statement on whether measurements were taken from distinct samples or whether the same sample was measured repeatedly
☐	☒	The statistical test(s) used AND whether they are one- or two-sided<br>*Only common tests should be described solely by name; describe more complex techniques in the Methods section.*
☒	☐	A description of all covariates tested
☐	☒	A description of any assumptions or corrections, such as tests of normality and adjustment for multiple comparisons
☐	☒	A full description of the statistical parameters including central tendency (e.g. means) or other basic estimates (e.g. regression coefficient) AND variation (e.g. standard deviation) or associated estimates of uncertainty (e.g. confidence intervals)
☐	☒	For null hypothesis testing, the test statistic (e.g. *F*, *t*, *r*) with confidence intervals, effect sizes, degrees of freedom and *P* value noted<br>*Give P values as exact values whenever suitable.*
☒	☐	For Bayesian analysis, information on the choice of priors and Markov chain Monte Carlo settings
☒	☐	For hierarchical and complex designs, identification of the appropriate level for tests and full reporting of outcomes
☐	☒	Estimates of effect sizes (e.g. Cohen's *d*, Pearson's *r*), indicating how they were calculated

Our web collection on statistics for biologists contains articles on many of the points above.

Software and code

Policy information about availability of computer code

Data collection

Lidomics data for LTP were acquired on an Agilent 1260 HPLC system coupled to a Q-Exactive Plus (Thermo) operated by a Xcalibur software versions 2.5.0.2042 or 2.8.1.2806. Lipidomics data for LTP gain-of-function was performed by Lipotype GmbH (Dresden, Germany) on a QExactive mass spectrometer (Thermo Scientific) equipped with a TriVersa NanoMate ion source (Advion Biosciences). SEC experiments were performed on an Ettan LC (GE Healthcare) or an Akta Pure (Cytiva) systems. Imaging of WB and HTPLC was done on a ChemiDoc (Bio-Rad) and a Pharos FX Plus molecular imager (Bio-Rad) imaging systems. Fluorescent-based binding assay was performed on a Spectramax Paradigm plate reader Molecular Devices piloted by SoftMax Pro 7 software.

Data analysis

Raw LC-MS/MS data were converted to mzML format with MS Convert from the ProteoWizard package (version 3.0.679). Peak picking, feature detection and alignment were done with PEAKS Studio 7.0 (Bioinformatics Solutions Inc.). HPTLC data were analysed using ImageJ 1.48m. Analysis of the fluorescent-based lipid binding data were performed using Graphpad Prism 10 version 10.6.1. The data analyses were done with R version 3.5.0 on a x86_64-w64-mingw32 platform. Scripts were tested with R version 4.2.2 on a x86_64-w64-mingw32/x64 (64-bit) platform. The following packages (algorithms) were used: for the visualization of beanplots, we employed the R package beanplot (version 1.2); for the circular visualization and heatmaps, we employed the packages circlize (version 0.4.12) and ComplexHeatmap (Bioconductor, version 2.3.2); for various string modifications, we employed the stringr package (version 1.4.0); for coloring schemes and gradients, we employed the RColorBrewer package (version 1.1-2); to extract input from Uniprot, we used the UniprotR package (version 1.2.4.); for automatic structural chemical entity classification we used ClassyFire (http://classyfire.wishartlab.com/). Identification and calculations of the volumes of the LTP pockets was done using the voronota-pocket script in the Voronota software package (version 1.28.4083) available at https://github.com/kliment-olechnovic/voronota/releases/tag/v1.28.4083. The volume of the lipids required an automated calculation of the number and nature of atoms and bonds, which was done with the RDKit: Open-Source Cheminformatics Software. Systems for molecular dynamics simulations were prepared using the CHARMM-GUI web server, the EDock web server, and the CHARMM General Force Field

(CGenFF) program version 2.5.1. The CGenFF force field v. 4.6 and the CHARMM36m force field with its extension for WYF-choline cation-π interactions were used. Molecular dynamics simulations were conducted using the NAMD3 simulation package. The analyses were conducted using several software: Charmm (version 49a1), an in-house code based on the MDAnalysis package (https://github.com/reuter-group/MD-contacts-analysis), and VMD. PyMol (version 3.0.0) and VMD (version 1.9.3) were used to prepare figures of protein structures.
The codes are accessible via in https://github.com/saezlab/lipyd and https://github.com/krtiteca/ScriptsAssociatedWithLTPArticle. The voronota-pocket script is available at https://github.com/kliment-olechnovic/voronota/blob/master/voronota-pocket. The in-house code based on the MDAnalysis package is available at https://github.com/reuter-group/MD-contacts-analysis.

For manuscripts utilizing custom algorithms or software that are central to the research but not yet described in published literature, software must be made available to editors and reviewers. We strongly encourage code deposition in a community repository (e.g. GitHub). See the Nature Portfolio guidelines for submitting code & software for further information.

Data

Policy information about availability of data

All manuscripts must include a data availability statement. This statement should provide the following information, where applicable:

- Accession codes, unique identifiers, or web links for publicly available datasets
- A description of any restrictions on data availability
- For clinical datasets or third party data, please ensure that the statement adheres to our policy

The lipidomics data can be downloaded from https://www.ebi.ac.uk/metabolights/MTBLS9567. Organelle lipidomics data are available at https://doi.org/10.1101/2025.10.05.680593.
The source data are organized as followed: molecular biology and LTP expression, MS fragmentation behaviour Supplementary Tables 1,2 and 3, respectively; lipid species or subclasses bound to LTP, Supplementary Tables 4 and 5, respectively; LTPs lipidated in only one of the assays, Supplementary Table 6; Results of the structural and functional benchmarks, Supplementary Tables 7 and 8; Functional relationship of lipids co-mobilized by the same LTP, Supplementary Table 9; Lipidomic data of bovine liver / brain extracts and HEK293 cells, Supplementary Table 10; Lipidomics CERT-over-expressing HeLa cells, Supplementary Table 11. Supplementary Tables used to produce the panels of the figures are summarized in Supplementary Table 14. Gels, SEC profiles and western blots are provided in Supplementary Figure 1.
External datasets analysed (but not generated) in this work are: sequences of human LTPs, UniProtKB (https://www.uniprot.org/uniprotkb) and SMART (https://smart.embl.de/smart/change_mode.cgi); definition of domains and motifs, InterPro (https://www.ebi.ac.uk/interpro/) and PFAM (http://pfam.xfam.org/); lipid identification rule-sets, SwissLipids (https://swisslipids.org/) and METLIN (https://metlin.scripps.edu/); SMILES for 1D lipid representations, LIPID MAPS (https://www.lipidmaps.org/); analyses of HSDL2 protein interactome, STRING (https://string-db.org/); analyses of organelle targeting sequences, PSORT (https://psort.org/) and PROSITE (https://prosite.expasy.org/); coregulated lipids, https://doi.org:10.1016/j.cell.2015.05.051; lipid colocalization, METASPACE (https://metaspace2020.org/); lipid subcellular localisation, https://doi.org:10.1101/2025.10.05.680593; protein structures, PDB (https://www.rcsb.org/); simulated protein structures, AlphaFold DB (https://alphafold.ebi.ac.uk/). External datasets analysed (but not generated) in this work are summarized in Supplementary Table 15.

Research involving human participants, their data, or biological material

Policy information about studies with human participants or human data. See also policy information about sex, gender (identity/presentation), and sexual orientation and race, ethnicity and racism.

Reporting on sex and gender	We have not performed research involving human participants
Reporting on race, ethnicity, or other socially relevant groupings	We have not performed research involving human participants
Population characteristics	We have not performed research involving human participants
Recruitment	We have not performed research involving human participants
Ethics oversight	We have not performed research involving human participants

Note that full information on the approval of the study protocol must also be provided in the manuscript.

Field-specific reporting

Please select the one below that is the best fit for your research. If you are not sure, read the appropriate sections before making your selection.

☒ Life sciences ☐ Behavioural & social sciences ☐ Ecological, evolutionary & environmental sciences

For a reference copy of the document with all sections, see nature.com/documents/nr-reporting-summary-flat.pdf

Life sciences study design

All studies must disclose on these points even when the disclosure is negative.

Sample size	No calculations were performed to determine the sample size. The sample size was limited by the number and availability of human LTP cDNAs and our success in expressing LTP in HEK293 and E. coli, respectively.

Data exclusions	No data were excluded.
Replication	All AP-MS analyses were performed once with lipidomic analyses of at least three adjacent SEC fractions in positive and negative ionization modes. Lipidomic analyses of HEK293 and HeLa cells overexpressing or not overexpressing LTP were performed in three independent biological replicates. The fluorescence emission shift binding assay was independently repeated eight times. All simulations were performed in triplicate, using a different velocity distribution for each replicate, and yielded similar results.
Randomization	Samples were not allocated to experimental groups. The HEK293-LTP cells that were cultured in parallel (batches) were selected so that they belonged to different LTP families (different names) and so that the molecular weight of the overexpressed LTPs was different in order to avoid any exchange of cell lines and to make any errors visible. For AP-MS experiments, samples were processed for LC-MS/MS as soon as they became available. For lipidomic analyses of LTP-overexpressing lines, they were randomized using the Rand() function in Microsoft Excel.
Blinding	Differences in the growth rates between the different cell lines and the need to verify that HEK293 and E. coli overexpressed LTP with the correct molecular weight prevented investigator blinding. Investigators were blinded to sample identity for the lipidomics analyses (AP-MS and HEK293 cells over-expressing LTP).

Reporting for specific materials, systems and methods

We require information from authors about some types of materials, experimental systems and methods used in many studies. Here, indicate whether each material, system or method listed is relevant to your study. If you are not sure if a list item applies to your research, read the appropriate section before selecting a response.

Materials & experimental systems

n/a	Involved in the study
☐	☒ Antibodies
☐	☒ Eukaryotic cell lines
☒	☐ Palaeontology and archaeology
☒	☐ Animals and other organisms
☒	☐ Clinical data
☒	☐ Dual use research of concern
☒	☐ Plants

Methods

n/a	Involved in the study
☒	☐ ChIP-seq
☒	☐ Flow cytometry
☒	☐ MRI-based neuroimaging

Antibodies

Antibodies used	anti-HA, 12CA5 mouse hybridoma, EMBL PEPCF (from InVivo BioTech Services, GmbH a Bruker company, clone name 12CA5; batch AK2055/01B.1); dilution 1:1.000); Anti-Mouse IgG HRP, cat. nr GENA931-1ML, Sigma-Aldrich (Cytiva)(dilution 1:10.000) .
Validation	All antibodies were validated by the manufacturer for their suitability for use in western blot applications.<br>Anti-HA, 12CA5 -> Applications: dot blots, immunochemistry[, immunoprecipitation, western blotting<br>Anti-Mouse IgG HRP, GENA931 -> Applications: mouse IgG HRP linked whole Ab has been used in immunoblotting

Eukaryotic cell lines

Policy information about cell lines and Sex and Gender in Research

Cell line source(s)	Flp-In T-REx-293 (Cat.Nr R78007) and Flp-In T-REx-HeLa (Cat.Nr R71407) were sourced from Thermofisher.
Authentication	No further authentication was performed.
Mycoplasma contamination	These cells were regularly checked and tested negative for mycoplasma.
Commonly misidentified lines<br>(See ICLAC register)	No commonly misidentified cell lines were used in this study.

Plants

Seed stocks	We did not use seed stockes
Novel plant genotypes	We do not report novel plant genotypes
Authentication	We have not done plant authentication
